# Dynamic modulation of genomic enhancer elements in the suprachiasmatic nucleus, the site of the mammalian circadian clock

**DOI:** 10.1101/gr.277581.122

**Published:** 2023-05

**Authors:** Akanksha Bafna, Gareth Banks, Michael H. Hastings, Patrick M. Nolan

**Affiliations:** 1 Medical Research Council, Harwell Science Campus, Oxfordshire OX11 0RD, United Kingdom;; 2 MRC Laboratory of Molecular Biology, Cambridge Biomedical Campus, Cambridge CB2 0QH, United Kingdom

## Abstract

The mammalian suprachiasmatic nucleus (SCN), located in the ventral hypothalamus, synchronizes and maintains daily cellular and physiological rhythms across the body, in accordance with environmental and visceral cues. Consequently, the systematic regulation of spatiotemporal gene transcription in the SCN is vital for daily timekeeping. So far, the regulatory elements assisting circadian gene transcription have only been studied in peripheral tissues, lacking the critical neuronal dimension intrinsic to the role of the SCN as central brain pacemaker. By using histone-ChIP-seq, we identified SCN-enriched gene regulatory elements that associated with temporal gene expression. Based on tissue-specific H3K27ac and H3K4me3 marks, we successfully produced the first-ever SCN gene-regulatory map. We found that a large majority of SCN enhancers not only show robust 24-h rhythmic modulation in H3K27ac occupancy, peaking at distinct times of day, but also possess canonical E-box (CACGTG) motifs potentially influencing downstream cycling gene expression. To establish enhancer–gene relationships in the SCN, we conducted directional RNA-seq at six distinct times across the day and night, and studied the association between dynamically changing histone acetylation and gene transcript levels. About 35% of the cycling H3K27ac sites were found adjacent to rhythmic gene transcripts, often preceding the rise in mRNA levels. We also noted that enhancers encompass noncoding, actively transcribing enhancer RNAs (eRNAs) in the SCN, which in turn oscillate, along with cyclic histone acetylation, and correlate with rhythmic gene transcription. Taken together, these findings shed light on genome-wide pretranscriptional regulation operative in the central clock that confers its precise and robust oscillation necessary to orchestrate daily timekeeping in mammals.

Most organisms possess intrinsic circadian (∼1-d) clocks that align their molecular, behavioral, and physiological processes to changing daily environmental conditions. In mammals, the phase and amplitude of circadian processes are directed and synchronized by the suprachiasmatic nucleus (SCN) of the hypothalamus, which facilitates robust 24-h oscillations in peripheral tissues (Schibler et al. 2015; Hastings et al. 2018). In turn, the SCN receives retinal input that ensures its autonomous circadian time is synchronized to solar time. Circadian time is generated by a genetic network of cell-autonomous transcription–translation feedback loops (TTFLs) driven by the activators circadian locomotor output cycles kaput (CLOCK) and basic helix-loop-helix ARNT like 1 (BMAL1) and the repressors period (PER) and cryptochrome (CRY) (Takahashi et al. 2008). At the circuit level, intercellular synchrony in the SCN is maintained through neuropeptidergic systems such as vasoactive intestinal peptide (VIP) and arginine vasopressin (AVP), which serve as coupling factors (Herzog et al. 2017). Ablation of the SCN leads to perpetual loss of circadian timing with out-of-sync peripheral clocks (Rusak and Zucker 1979; Yoo et al. 2004), whereas genetic restoration of the SCN TTFL has been shown to initiate 24-h rhythmicity at the molecular and behavioral levels (Maywood et al. 2021).

The TTFL is also active in peripheral tissues and cells but is far less robust than that of the SCN, typically damping after a few cycles, ex vivo, whereas the SCN can continue to oscillate indefinitely in explant culture. This raises the question that apart from the distinctive intra- and inter-cellular coupling, what features of transcriptional architecture render the SCN such a powerful clock. We hypothesize that precise regulation of spatiotemporal gene expression in the SCN is imperative for its powerful daily timekeeping. Daily rhythms of electrical activity, metabolic rate, and intra- and inter-cellular signaling are driven by cascades of transcription, co-ordinated by clock-controlled genes (CCGs), many of which are transcription factors (TFs) sensitive to the TTFL. Such rhythmic gene transcription depends on the sequential co-ordination of multiple layers of epigenetic events, ranging from chromatin accessibility to TF binding (Yeung et al. 2018). The regulatory enhancer–promoter interactions have been shown to govern RNA polymerase II (RNAP II) recruitment followed by TF and cofactor binding, leading to cell- and tissue-specific expression through the initiation of mRNA transcription. In the mouse liver, for example, 24-h periodic occupancy by chromatin remodeling enzymes permits RNAP II and BMAL1 recruitment to support local circadian gene expression (Koike et al. 2012; Sobel et al. 2017). Additionally, advances in genome sequencing technologies have highlighted the occurrence of rhythmic modulation in chromatin topology that facilitates proximal and distal circadian gene expression (Aguilar-Arnal et al. 2013; Kim et al. 2018; Mermet et al. 2018) through promoter–enhancer “looping.”

Recently, active enhancer sites have also been reported to transcribe enhancer RNA (eRNA) dynamically in response to chemical and electrical stimuli, learning, and behavioral experiences (Kim et al. 2010; Telese et al. 2015; Joo et al. 2016). This class of noncoding RNAs are generally not spliced or polyadenylated (Arner et al. 2015; Gray et al. 2015) and are believed to assist long distance enhancer–promoter interaction. Incidences of circadian eRNAs oscillating and peaking with distinct phases have been observed in the mouse liver transcriptome (Fang et al. 2014). Such circadian variation in abundance of eRNA adds an important layer of control in the regulation of gene expression in defined time and space.

At the tissue level, the persistence and precision of the SCN as a circadian timekeeper are unique, but the cell-autonomous regulatory factors that confer this uniqueness to the SCN are poorly understood. Genomic mapping of regions with associated histone modifications such as H3K4me3 and H3K27ac has been the cornerstone for identifying active promoter and enhancer sites, respectively (Heintzman et al. 2007; Won et al. 2008). Typically, during active transcription, looser chromatin structures facilitate binding of TFs and RNA polymerases to trigger target gene expression. For instance, acetylation of a lysine residue of H3 histone weakens the binding between the histone and negatively charged DNA and exposes the DNA to regulatory proteins (Dong and Weng 2013; Starks et al. 2019). Trimethylation of the lysine at the fourth position of the H3 histone is another conspicuous modification that is seen largely near transcription start sites (TSSs) of the transcribing genes, marking the promoter regions.

Here, we used histone chromatin immunoprecipitation (ChIP) sequencing (histone ChIP-seq) to map genome-wide gene promoter and active enhancer sites in the SCN. Next, with a view to identifying putative SCN-enriched regulatory processes, we compared the identified histone modification peak profiles in the SCN with those found in the cerebral cortex. This revealed considerable enrichment associated with genes highly expressed in the SCN (Brown et al. 2017), supporting the view that histone modifications are reliable markers for mapping gene regulatory elements in a locus-, cell-, and tissue-specific manner (Garcia et al. 2008; Ngo et al. 2019). We then turned our attention to the 24-h oscillatory patterns at the identified gene regulatory *cis*-elements, which presumably are vital to drive the SCN circadian rhythm. Furthermore, we performed directional bulk RNA-seq at six distinct time points to study the relationship between observed rhythmic H3K27ac occupancy and target gene expression. Along with rhythms in the coding mRNA fraction, we also noticed cycling expression in the noncoding bidirectional eRNA. Thereby, our study offers a framework to understand the dynamic pretranscriptional regulation operative in the SCN central clock that potentially contributes to maintaining its uniquely stable circadian timekeeping.

## Results

### Genome-wide promoter and enhancer site mapping in the SCN using histone ChIP-seq

**Figure 1.**
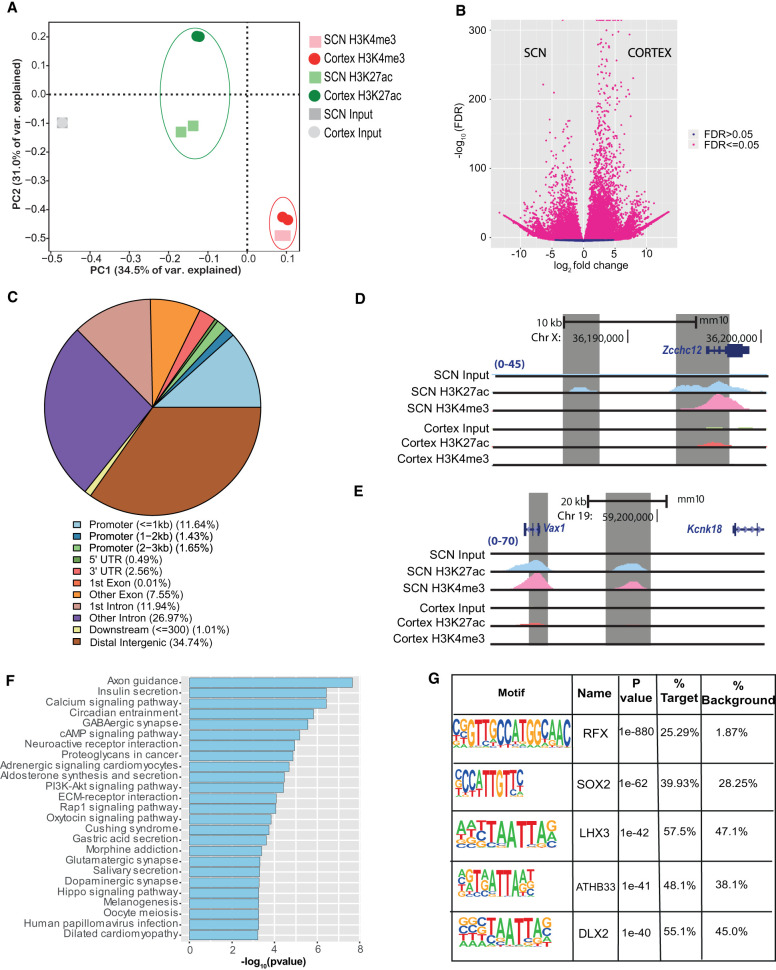
Genome-wide characterization of SCN enhancers. (*A*) PCA analysis of aligned reads post H3K4me3 and H3K27ac immunoprecipitation from the SCN and cortex mouse brain tissues at ZT3. (*B*) Volcano plot showing fold change and false-discovery rate (FDR) for differential H3K27ac sites between the SCN and cortex as computed by Diffbind. (*C*) Genomic feature distribution of SCN-enriched H3K27ac peaks (n = 14,153). (*D*,*E*) UCSC Genome Browser tracks showing histone modifications H3K4me3 and H3K27ac normalized ChIP-seq read coverage (shaded gray) along with their input for the SCN and cortex at representative examples. The chromosome location and scale (mm10 genome) are indicated at the *top*. (*F*) Functional annotation of nearest neighboring gene (TSS) to SCN-enriched H3K27ac sites (fold change > 5) using the KEGG pathway (DAVID). (*G*) Overrepresented transcription factor binding motifs using SCN-enriched H3K27ac sites as target by HOMER.

To identify DNA regulatory elements influencing gene transcription in the SCN, we focused on SCN-enriched promoter and enhancer sites marked by H3K4me3 and H3K27ac, respectively. C57BL/6J mice were kept in standard 12-h/12-h light–dark conditions, and eight animals per time point (Methods) were used for subsequent brain dissections. The harvested SCN and cerebral cortical brain regions were then used for H3K4me3 and H3K27ac ChIP-seq, and the resulting sequencing reads were mapped to the mouse genome (Methods). Initial principal component analysis (PCA) of aligned reads arising from H3K4me3 and H3K27ac occupancy at ZT3 confirmed high-quality ChIP signal and tissue-specific enrichment. The reads associated with H3K4me3 and H3K27ac, respectively, not only were found to be separate from their corresponding input sample but also showed clear distinction based on the brain region (Fig. 1A). In addition, the variability between tissue-specific (SCN vs. cortex) H3K27ac marks was greater than the H3K4me3 counterpart, suggesting a potential role for active enhancers marked by H3K27ac in defining brain tissue identity and function (Ko et al. 2017). As expected, the occupancy of H3K4me3 (along with H3K27ac) was highly abundant around TSSs (TSS ± 2 kb), presumably arising from the actively transcribing genes (Supplemental Fig. S1; Beacon et al. 2021). A collection of 10,577 H3K4me3 peaks (FDR ≤ 0.05) was observed to differ in abundance significantly between the SCN and cortex, with the majority located in the promoter region (Supplemental Fig. S2A,B; Supplemental Table S1). The SCN-enriched H3K4me3 sites were found adjacent to TSSs of the genes known to be highly expressed in the tissue, as exemplified in Supplemental Figure S2C (Brown et al. 2017; Wilcox et al. 2017), whereas many were associated with synaptic processes and signaling pathways (Supplemental Fig. S2D).

**Figure 2.**
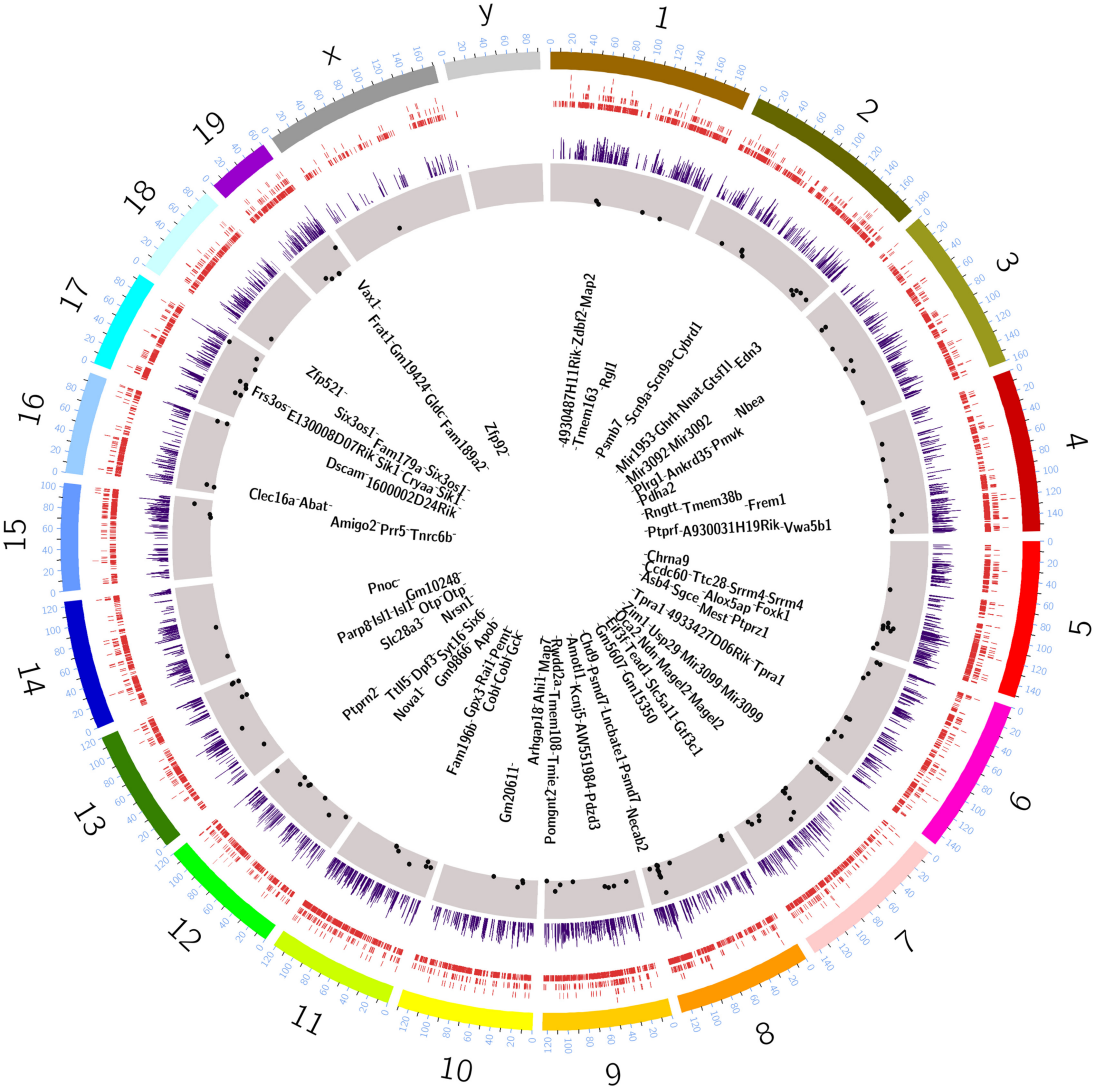
SCN-enhancer map. Circos plot (Krzywinski et al. 2009) for SCN-enriched enhancers with mm10 genome assembly and four layers (*outer* to *inner*). (Layer 1) SCN-enriched H3K27ac sites (n = 14,153). (Layer 2) Histogram showing SCN-enriched H3K27ac sites with fold change > 5 in comparison to the cortex. (Layer 3) Scatter plot showing subselected SCN-enriched H3K27ac sites with fold change > 10. (Layer 4) Nearest gene (TSS) to the sites mapped in layer 3.

Having identified SCN-specific promoter marks via H3K4me3, we then sought to delineate active enhancers by mapping H3K27ac binding. We identified approximately four times more H3K27ac peaks (44,247 sites, FDR ≤ 0.05) that differentially marked active enhancer sites between the two brain regions (Fig. 1B; Supplemental Table S2A). These SCN-enriched H3K27ac sites were dominant at distal intergenic (34.7%) and intronic regions, including first introns (38.9%) (Fig. 1C), which is consistent with previous reports (Creyghton et al. 2010; Rada-Iglesias et al. 2011) wherein tissue-specific enhancer marks were seen to be highly abundant at intergenic and intragenic regions. Next, we preferentially selected highly distinct (fold change ≥ 5, n = 4592) (Fig. 1D,E) SCN-specific H3K27ac sites and recorded the closest TSS (±3 kb), guided by the peak annotation tool ChIPseeker (Methods). Many of the genes observed to be adjacent to these active enhancer marks were found to be implicated in SCN-enriched functions, including circadian entrainment, calcium signaling, and GABAergic synaptic function (Fig. 1F; Koronowski and Sassone-Corsi 2021; Morris et al. 2021). Furthermore, motif analysis on H3K27ac-bound regions (Methods) revealed enrichment of the RFX and SOX families of TF binding sites that are known to be present in adult SCN tissue and influence light entrainment pathways (Fig. 1G; Araki et al. 2004; Cheng et al. 2019). Overall, we produced a genome-wide distribution of SCN-enhancer marks (Fig. 2, layer 1–3) along with the neighboring target gene (layer 4), which likely supports the role of the SCN as the principal circadian pacemaker. The SCN-enriched enhancer sites are well distributed across the genome, with a high incidence of the enriched sites (fold change > 10) (Fig. 2, layer 3) at certain genomic locations such as Chromosome (Chr) 7 and Chr 17. Many of these SCN-enriched enhancers were also found clustered together, as seen within the *Usp29* (±0.2 Mb) gene at Chr 7 and the *Zfhx3* (±1 Mb) gene at Chr 8. It is noteworthy that the SCN TF ZFHX3 has been implicated as an important regulator in setting the robustness and speed of the circadian clock (Parsons et al. 2015), and the prevalence of SCN-enriched enhancer marks hints on its tissue-specific transcriptional regulation.

### Time-of-day-dependent variation in enhancer activity in the SCN

**Figure 3.**
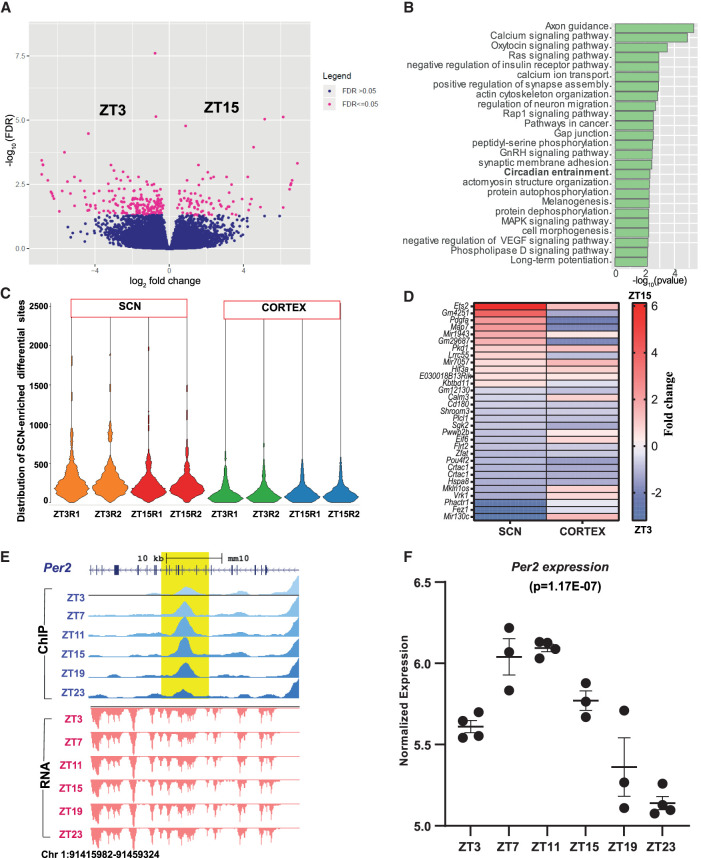
Dynamic H3K27ac occupancy based on time of the day. (*A*) Volcano plot showing fold change and FDR for differential H3K27ac sites in the SCN between ZT3 and ZT15 (n = 293) as computed by Diffbind. (*B*) Functional annotation of nearest gene (TSS) from differential (ZT3 vs. ZT15) H3K27ac sites using GO (biological process) and KEGG pathway (DAVID). (*C*) Violin plot showing distribution of differential H3K27ac peaks at ZT3 and ZT15 in the SCN and cortex: (R1) replicate 1, (R2) replicate 2. (*D*) Heat map view of differential H3K27ac sites (ZT3 vs. ZT15) observed in both the SCN and cortex. (*E*) UCSC Genome Browser tracks for H3K27ac intragenic peak (blue with shaded yellow) at the *Per2* gene locus showing ZT11 and ZT23 differential peak (fold change = 1.7) along with the gene expression tracks (red) across six time points (ZT3–ZT23) arising from the negative strand. (*F*) Normalized gene expression level of the *Per2* gene across six distinct time points, cycling with a peak at ZT11 and a trough at ZT23; Pearson's correlation (H3K27ac peak and gene expression) = 1.

The potential regulation of the abundance of histone modifications (H3K4me3 and H3K27ac) was then investigated by comparing the antiphasic (12-h-apart) time points, ZT3 (3 h after light on) and ZT15 (3 h after light off), in both the SCN and cerebral cortex. We observed approximately 551 differential H3K4me3 sites in the cortex at FDR ≤ 0.05 (Supplemental Fig. S3A; Supplemental Table S3A), mostly confined around the TSS of genes involved in learning and synapse organization. In contrast, no changes in H3K4me3 sites were seen within the SCN (Supplemental Table S3B). This stability of histone trimethylation levels in the SCN is in marked contrast to studies in peripheral clock(s) (Koike et al. 2012; Le Martelot et al. 2012; Valekunja et al. 2013), wherein occupancy of the promoter mark was shown to be cyclic. Rhythmic trimethylation may therefore be a conserved feature of subordinate, SCN-driven clocks in the cortex and peripheral tissues. In contrast, there was strong differential H3K27ac occupancy within the SCN between day (ZT3) and night (ZT15; n = 293) (Fig. 3A; Supplemental Table S4A). This reinforces the potential role of H3K27ac-marked active enhancers, as opposed to H3K4me3 marks, in regulating time-of-day-dependent gene transcription. On further inspection, a significant proportion of identified differentially H3K27ac-marked enhancer sites was found proximal to genes implicated in circadian entrainment, such as *Gnas*, *Rasd1*, and *Cacna1a* (Fig. 3B; Supplemental Table S3C,D; Brown et al. 2017; Harvey et al. 2020). Therefore, we could precisely identify time-of-day-dependent H3K27ac variance in the SCN as a potential contributor to, and/ or output from, daily TTFL timekeeping. We then examined whether the observed differential H3K27ac sites in the SCN are also present in the cortex, even though they may not be time dependent (ZT3 vs. ZT15). About half of these differential sites (n = 146) revealed greater H3K27ac abundance in the SCN (Fig. 3C; Supplemental Fig. S3B), with almost negligible occurrence in the cortex. Moreover, active enhancer sites that showed day–night fluctuation in both the SCN and cortex (n = 31) did not follow the same trend; for example, a differential H3K27ac site mapped to calcium binding gene *Calm3* is strongly elevated at ZT3 in the SCN but is elevated at ZT15 in the cortex (Fig. 3D), where it may potentially control antiphasic peaks of gene expression (Chun et al. 2015). Hence, this difference in H3K27ac occupancy suggests a role of *cis*-regulatory elements in directing the tissue-specific gene transcriptional machinery in response to environmental and cell-autonomous stimuli. It also highlights the possibility of the same enhancer site being active at a distinct time of day to regulate the subsequent tissue-specific gene expression.

To understand further the dynamics of H3K27ac occupancy in the context of daily timekeeping in the SCN, we profiled its occurrence every 4 h starting from ZT3 (Methods) and compared the peak profiles between additional antiphasic time points (ZT7 vs. ZT19, ZT11 vs. ZT23), based on the expectation that rhythmic genes under circadian regulation would have peaks and troughs in expression level ∼12 h apart (Supplemental Fig. S3C). Indeed, we found a considerable number of active enhancer sites in the SCN (Supplemental Table S4B,C) with differential abundances at distinct times of day. In addition, there was almost negligible overlap between H3K27ac-marked differential sites noticed from distinct antiphasic comparisons; for instance, an enhancer peak differential between ZT3 and ZT15 did not overlap with any found between ZT7 and ZT19 (i.e., events were temporally segregated). Using this approach, we clearly identified a unique active enhancer site within the *Per2* gene showing increased histone acetylation at ZT11 in comparison with ZT23, coinciding with the peak and trough of host gene expression levels (Fig. 3E,F). Similarly, a differential H3K27ac peak (ZT7 vs. ZT19) observed upstream of the *Cry1* TSS (Chr 10: 85,182,849–85,186,296) is concordant with the downstream gene transcription (Supplemental Table S4B). Thus, variability in histone acetylation levels seems to be clearly associated with changing transcriptional output in the SCN, as noted with the rhythmic expression of the core-clock genes *Per2* and *Cry1*. About 50% of the differing histone acetylation levels perfectly correlated (Pearson's correlation (*r*) = 1) with the host gene expression (Supplemental Fig. S4E). To our knowledge, this method of strategic in-depth examination in the time-dependent occurrence of histone modifications has never been reported previously. We clearly observed the time-dependent differing histone acetylation levels arising from TTFL components to downstream CCGs in the SCN.

Motif analysis of antiphasic differential H3K27ac sites revealed the predominance of distinctive TFs as a function of the time of day (Supplemental Fig. S3D). TF binding sites for CAAT/enhancer-binding protein (C/EBP) alpha (CEBPA) and RAR-related orphan receptor (ROR), both belonging to the PARbZip family (Gachon 2007), were abundant in the ZT3 versus ZT15 comparison, whereas those for NF-kB, implicated in the mammalian circadian clock, were enriched in the ZT11 versus ZT23 comparison (Shen et al. 2021). This is consistent with the finding in the liver in which phase-separated functional enhancers were driven by distinct TFs (Fang et al. 2014). Therefore, we assume that the change in histone acetylation is important for the timely binding of TFs to drive temporal gene expression as noted across various tissues.

### Diurnal variation in H3K27ac occupancy linked to rhythmic gene transcription in the SCN

**Figure 4.**
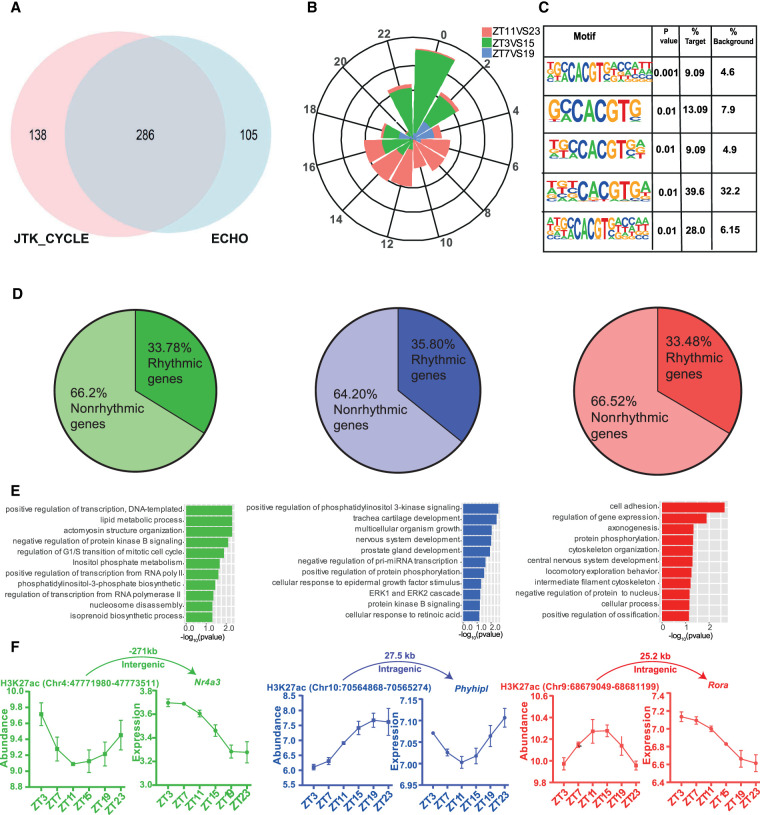
Rhythmic H3K27ac occupancy linked to cycling gene expression in the SCN. (*A*) Venn diagram showing overlap of rhythmic H3K27ac abundance as analyzed by ECHO and JTK_CYCLE in the SCN. (*B*) Phase distribution of cycling H3K27ac peaks that were found differential in ZT3 versus ZT15 (green), ZT7 versus ZT19 (blue), and ZT11 versus ZT23 (red) groups denoted by a rose plot. (*C*) Overrepresentation of the E-box motif (CACGTG) in oscillating H3K27ac sites (HOMER). (*D*) Proportion of rhythmic genes mapped adjacent to rhythmic H3K27ac regions for each phase separated enhancer group. (*E*) Functional annotation of rhythmic genes found in close proximity of cycling H3K27ac abundance, peaking at distinct time (ZT), using GO (biological process) and KEGG pathway (DAVID). (*F*) Representative examples of rhythmic H3K27ac and target gene expression (*P* < 0.05) with distance to TSS and genomic location shown at *top*.

To determine the prevalence of 24-h oscillations in H3K27ac abundance, we considered peaks that showed differential occupancy between any antiphasic (12-h-apart) comparisons. Active enhancer sites that were found to be distinct between these were compiled (n = 1021) and further assessed for circadian oscillation using the Extended Circadian Harmonic Oscillator (ECHO) application (De Los Santos et al. 2020). Briefly, logarithmic normalized counts per million (CPM) per peak interval was used across six distinct time points to determine 24-h oscillation (Methods). Of these, approximately half were recorded as rhythmic, and almost a quarter (n = 286) also showed robust oscillation (*P* ≤ 0.05) by both JTK_CYCLE (Hughes et al. 2010) and ECHO (Fig. 4A; Supplemental Fig. S4A; Supplemental Table S5). The canonical E-box (CACGTG) element facilitating circadian transcriptional activation by binding of CLOCK and BMAL1 (Ripperger and Schibler 2006) was overrepresented among the periodic H3K27ac sites (Fig. 4C). In principle, acetylation of the lysine residues of histones is known to promote binding of TFs by weakening histone-DNA interactions (Roth et al. 2001). Thus, the occurrence of rhythmic histone acetylation potentially triggers a wave of chromatin accessibility and facilitates binding of clock TFs (E-box) to regulate cyclic gene transcription. Indeed, we noticed ∼35% of genes proximal to rhythmic H3K27ac sites were strongly cyclic (JTK_CYCLE, *P* < 0.05), suggesting a pivotal role of *cis*-regulatory elements in the maintenance of rhythmic transcriptional output in the SCN (Fig. 4D,F; Supplemental Table S6). For example, RAR-related orphan receptor alpha (*Rora*) is known to regulate the rhythmic expression of *Bmal1* (Akashi and Takumi 2005), and it peaks following the periodic rise of nearby histone acetylation levels as seen in Figure 4F. This timely coordination in gene transcription provided by the adjacent histone modifiers highlights the importance of dynamic pretranscriptional regulation in the SCN. Considering the cycling gene expression in the SCN peaks at various times of day (Supplemental Fig. S4B; Supplemental Table S7), we also observed uniform phase (peak abundance) distribution for the rhythmic H3K27ac sites at the 24-h period (Fig. 4B). Although a significant proportion of these oscillating sites were found at intragenic regions, they were well separated from the corresponding promoters or TSSs, demarcating them from any H3K27ac (along with H3K4me3) signal arising directly owing to the host gene transcriptional activity (Supplemental Fig. S4C,D). For that reason, a high proportion of these rhythmic histone acetylation sites were found ± 50 kb from the rhythmic gene TSS. In addition, functional annotation of the rhythmic genes that are under the control of the phase-separated cycling H3K27ac levels in the SCN revealed diverse roles (Fig. 4E), ranging from initiation of transcription (ZT3 vs. ZT15; green plot) to protein phosphorylation (ZT11 vs. ZT23; red plot). To summarize, the observed robust oscillations in H3K27ac levels, peaking at distinct times of day, contribute to the cluster of rhythmic gene expression involved in distinct functions in the SCN. These rhythmic genes are either involved in the canonical TTFL or include downstream CCGs.

### De novo identification of actively transcribing eRNAs in the SCN

**Figure 5.**
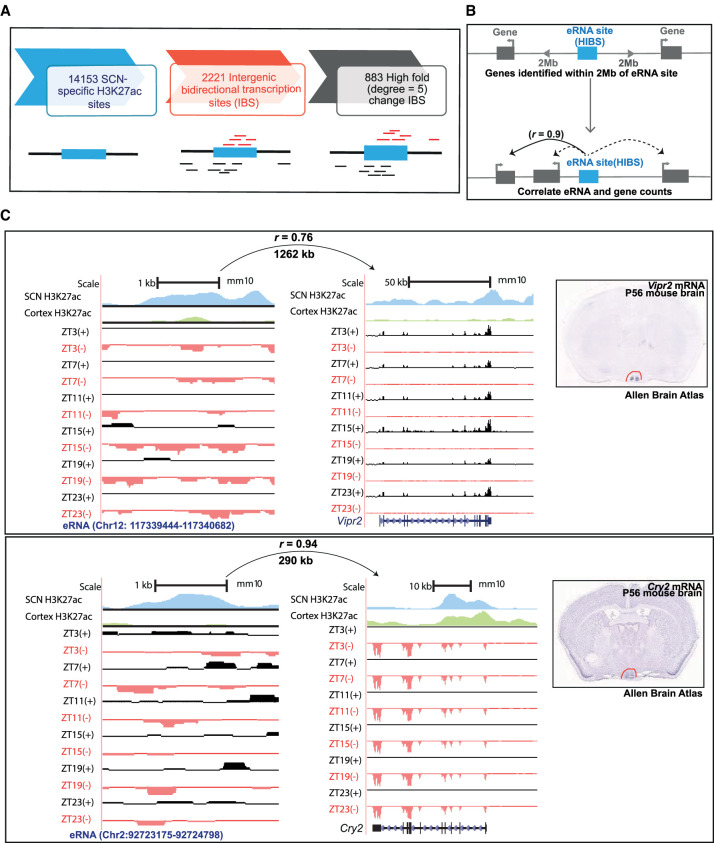
Identification of actively transcribing SCN enhancers (eRNAs). (*A*) Analysis pipeline for identification of actively transcribing enhancers. (*B*) eRNA–gene pairs were determined by correlation of eRNA and gene expression levels within a 2-Mb distance cutoff. (*C*) UCSC Genome Browser tracks showing enhancer-linked H3K27ac signal (normalized ChIP-seq coverage) for cortex (green) and SCN (blue) peaks and total RNA expression (reads mapped to + [black] and – [red] strand) signal as normalized CPM value for two representative examples, that is, *Vipr2* and *Cry2*. The distance between eRNA and predicted target gene with Pearson's correlation is indicated *above* each represented eRNA–gene pair. SCN expression of the respective target gene is also evident in in situ hybridization images from adult mouse brain (*right*; image credit: Allen Brain Atlas, Allen Institute).

We clearly identified approximately 14,153 (FDR ≤ 0.05) SCN-specific enhancer sites compared with the cortex (Fig. 2). A subset of SCN-enhancer marks, found at intergenic regions, were further investigated for bidirectional transcription, which is considered to be the hallmark of eRNA occurrence (Methods) (Fig. 5A). Unlike the coding mRNA component, these long noncoding fractions (median size = 346 nucleotides) are not spliced and are not polyadenylated (Andersson et al. 2014). With the recent advancement in high-throughput sequencing, the occurrence of eRNA along with H3K27ac histone modification has been recognized as a reliable measure of active enhancers and has been shown to regulate gene transcription in response to plasticity-inducing stimulation and behavioral experience (Malik et al. 2014; Telese et al. 2015). Therefore, using a direction-specific total RNA-seq SCN data set, we revealed 2221 intergenic bidirectional transcription sites (IBSs) (Supplemental Table S8). Out of these, we subselected IBSs that revealed greater SCN-specific H3K27ac occupancy (fold change > 5, HIBS) and focused on the high-confidence SCN actively transcribing enhancer sites (n = 883), as represented in Supplemental Figure S5, A and B. Based on previous findings, chromatin remodeling and eRNA transcription are shown to precede mRNA expression present at adjacent *cis*-loci (Schaukowitch et al. 2014; Arner et al. 2015; Kim et al. 2015). Therefore, the prevalence of eRNAs in the SCN presented an excellent opportunity to investigate its influence on gene transcriptional machinery.

Next, the expression of SCN-specific eRNA was evaluated at six distinct time points across the day starting from ZT3 (Methods). To determine the target gene under the control of each actively transcribing enhancer, we studied the correlation between eRNA and all annotated protein-coding gene expression falling within ∼±2 Mb from the peak center (Fig. 5B). This approach is based on the assumption that enhancers and linked genes will correlate in their transcriptional output, as previously observed across different cell classes or activity states (Carullo et al. 2020). Using positive correlation (*r* > 0.5), we noted 7795 high-confidence eRNA–gene pairs from a total of 46,022 identified pairs (Supplemental Table S9). As expected, the actively transcribing enhancers positively correlated with highly expressed SCN target genes as shown in the representative examples at the *Vipr2* and *Cry2* loci (Fig. 5C; Supplemental Fig. S5C). The distance of enhancer from the target gene had no effect on the subsequent strength of transcriptional correlation, consistent with the suggested role of eRNA in regulating both adjacent and distal gene expression (Supplemental Fig. S5D; Han and Li 2022). For example, an eRNA (Chr 4: 119,682,088–119,683,466) was found to be strongly correlated (*r* = 0.9) to both a 0.4-Mb distant target gene, prolyl 3-hydroxylase 1 (*P3h1*), and a 1.5-Mb separated gene, rearranged L-myc fusion (*Rlf*). Overall, we were clearly able to show the prevalence of noncoding eRNA in the SCN, arising from enriched histone acetylation sites, which are well placed to regulate local tissue-specific gene expression. Our data set pointed out toward the involvement of not just histone modifiers but also succeeding noncoding transcript in regulating the target gene transcription and prompted us to study its role in daily timekeeping.

### Circadian oscillations in identified eRNA expression in the SCN

**Figure 6.**
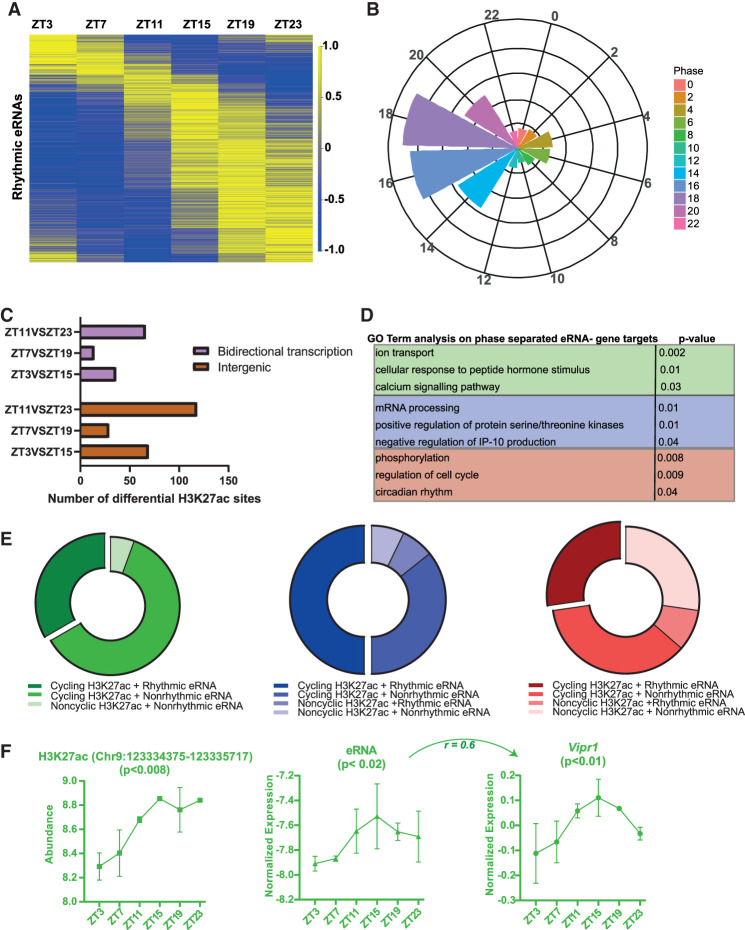
Differing H3K27ac peak abundance linked to rhythmic eRNA. (*A*) Heat map showing rhythmic eRNA expression throughout the day in the SCN. (*B*) Phase distribution of oscillating eRNA in 12 groups (2-h interval) depicted by radial plot. (*C*) Proportion of differential (antiphasic) H3K27ac sites present at intergenic regions showing bidirectional (eRNA) transcription. (*D*) Functional annotation of target genes controlled by eRNA arising from differential H3K27ac peak abundance at intergenic sites: ZT3 versus ZT15 (green), ZT7 versus ZT19 (red), and ZT11 versus ZT23 (blue). (*E*) Distribution of rhythmic and nonrhythmic SCN eRNAs arising from 24-h cyclic and/or differential H3K27ac sites between compared antiphasic group. (*F*) Representative example of rhythmic H3K27ac abundance, eRNA, and positively correlated gene expression (log CPM) as analyzed by ECHO (*P* < 0.05) with the indicated Pearson's correlation coefficient (r).

The identified eRNAs (Fig. 5B) from the SCN-enriched H3K27ac sites (HIBS; n = 883) were further examined for the occurrence of 24-h oscillation in expression levels. Similar to the cycling histone acetylation abundance, we found intensely rhythmic eRNAs (JTK_CYCLE, *P* < 0.05) (Fig. 6A; Supplemental Table S10) in the SCN, with their relative expressions peaking across different times of the day. Based on their phase of peak expression, circadian SCN eRNAs were divided into 12 groups (phase ZT0–ZT24, at 2-h intervals) (Fig. 6B). Circadian eRNAs did not cluster like the H3K27ac counterpart (Fig. 4B) or rhythmic mRNA (Supplemental Fig. S4B). Approximately 66% of circadian eRNAs oscillated with a peak phase between ZT14 and ZT20, whereas 34% oscillated in other phases. This irregular phase distribution of SCN eRNAs agrees with the previous finding wherein circadian eRNAs in liver peaked predominantly between ZT18 and ZT3 (Fang et al. 2014). Bearing in mind the central clock receives environmental stimuli and relays the signal to various peripheral clocks, the observed advanced phase peak in SCN circadian eRNAs relative to liver is consistent with the overall phases of their respective TTFLs. By and large, the rhythmic eRNAs peaking at distinct phases point toward the hierarchical regulation of the temporal gene expression in the SCN driven by functional enhancers.

### Differential H3K27ac occupancy coupled with rhythmic eRNA abundance in the SCN

To explore the link between differing histone acetylation at distinct times of day and eRNA expression levels, we assessed the intergenic class of differential H3K27ac sites (n = 216) for the occurrence of eRNA transcription. To accomplish this, strand-specific RNA abundance was examined for each intergenic site showing differential histone acetylation between antiphasic time intervals. About 53% of these intergenic H3K27ac sites revealed bidirectional transcription, a reliable measure for eRNA abundance (Fig. 6C; Supplemental Table S11). Subsequently, the expression level of each identified eRNA was examined across various times of the day for a positive correlation (*r* > 0.5) with the target gene transcription present at ∼±2-Mb distance (Supplemental Fig. S6A). Unlike the principal nearest neighboring gene approach, this strategy helped us to identify a repertoire of target genes whose expression is possibly regulated by distantly transcribing enhancers in the SCN (Supplemental Table S12). The recognized target genes were seen to be involved in divergent biological processes based on the disparate H3K27ac abundance as shown in Figure 6D. For example, eRNAs identified from differential H3K27ac sites between ZT11 and ZT23 seem to correlate and potentially regulate target genes (basic helix-loop-helix ARNT like [*Bmal1*]) (Supplemental Fig. S6B), prokineticin receptor 1 (*Prokr1*), etc., mediating circadian rhythms, whereas those identified from differential histone acetylation between ZT7 and ZT19 were found to influence genes involved in mRNA processing. Therefore, the noncoding eRNAs driven by temporally fluctuating histone acetylation levels add an important layer of control over the gene expression machinery in the SCN.

**Figure 7.**
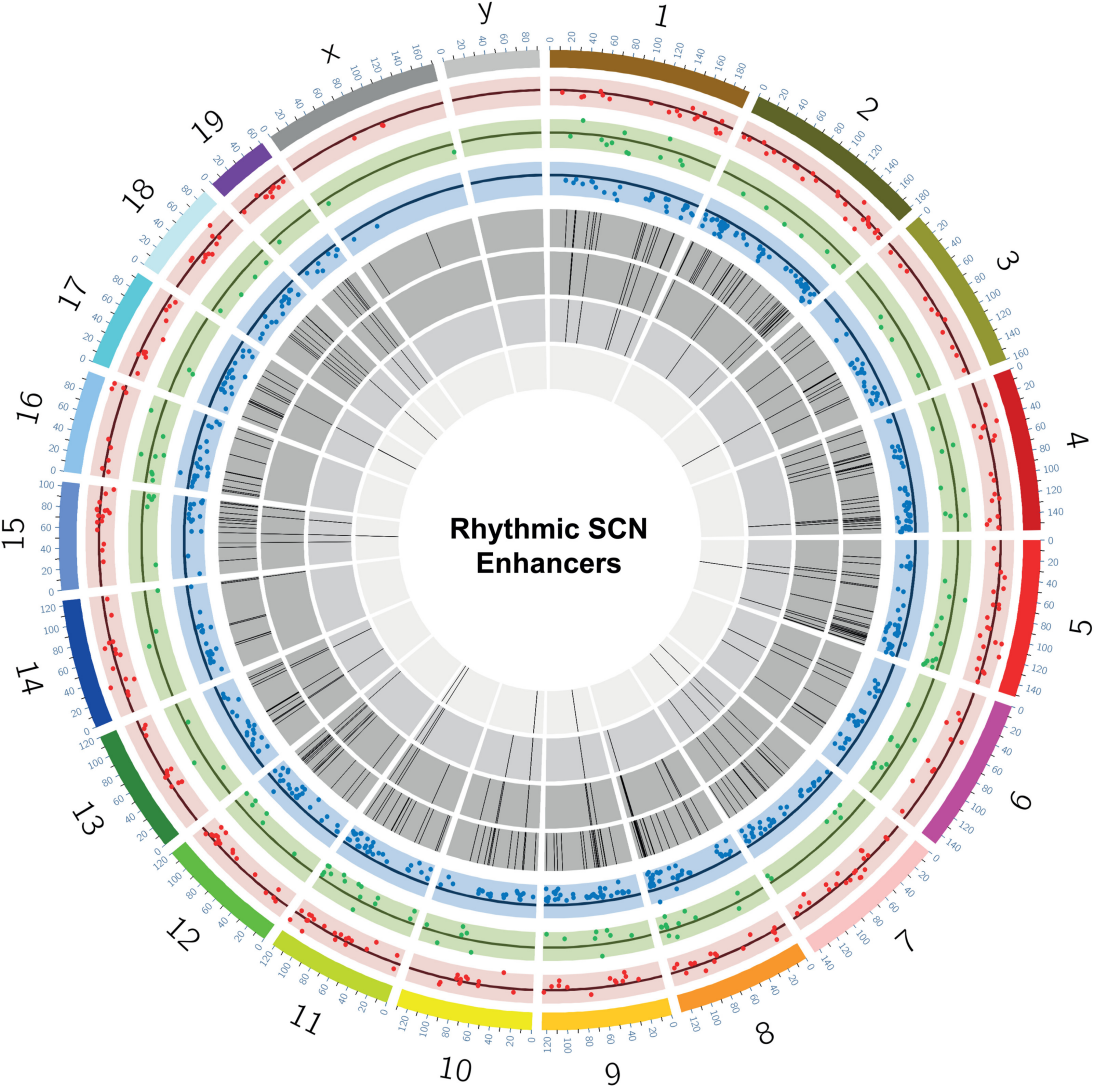
Snapshot of rhythmic H3K27ac abundance and overlapping eRNAs in the SCN. Circos plot (Krzywinski et al. 2009) for dynamic SCN-enriched enhancers with mm10 genome assembly and seven layers (*outer* to *inner*). (Layer 1) Differential H3K27ac sites as scatter plot (n = 293) between ZT3 and ZT15 (red). (Layer 2) Differential H3K27ac sites (n = 138) between ZT7 and ZT19 (green). (Layer 3) Differential H3K27ac sites (n = 582) between ZT11 and ZT23 (blue). (Layer 4) Histogram showing oscillating H3K27ac sites (n = 286; *P* < 0.05 both ECHO and JTK_CYCLE). (Layer 5) H3K27ac oscillating sites from layer 4 confined to intergenic position (n = 89). (Layer 6) eRNA (n = 37) arising from the intergenic H3K27ac sites highlighted in layer 5. (Layer 7) Subset of rhythmic eRNA (n = 12) coinciding with the rhythmic H3K27ac levels mapped at the intergenic sites.

Of note, ∼38% of these eRNAs arising from dynamic histone acetylation sites showed 24-h periodic transcription (ECHO/JTK_CYCLE, *P* < 0.05) (Fig. 6E). Thus, to our knowledge this is the first time in which rhythmic eRNAs were observed coupled with either cycling or differential (antiphasic) H3K27ac peaks (Supplemental Table S13). Taken together, we uncovered a proportion of intergenic sites with cycling H3K27ac levels (Fig. 7, see layer 7; Supplemental Fig. S7), which in turn transcribes rhythmic eRNA to regulate gene transcriptional machinery in the SCN. A characteristic example of this phenomenon is presented (Fig. 6F; Supplemental Fig. S6D), wherein rhythmic H3K27ac abundance and subsequent eRNA transcription (Chr 9: 123,334,375–123,335,717) are seen to regulate the circadian expression of the SCN neuropeptide receptor gene *Vipr1* (An et al. 2011). This was further confirmed by qPCR (Supplemental Fig. S6C), where the relative expression of varying eRNA directly coordinated with the target *Vipr1* mRNA transcription during the day. As expected, the eRNA and target *Vipr1* mRNA had high expression at ZT15 and low expression at ZT3, showing an in-phase relationship. Herewith, this comprehensive analysis of dynamic histone acetylation in conjunction with enhancer transcription helped us to unfold an important layer in the systematic regulation of circadian gene expression in the SCN.

## Discussion

The SCN is unique in its ability to sustain autonomous rhythmicity, which is driven by circadian cascades of transcription that direct metabolic, electrophysiological, and signaling rhythms. Our current finding highlights the instrumental role of gene regulatory elements in driving the daily timekeeping mechanism. In particular, we noted a dynamic pretranscriptional network, operative in the SCN, that potentially aids the rhythmic gene transcription and provides timely co-ordination to set the pace of the clock and confer robust high-amplitude rhythms. First, we profiled genome-wide posttranslational histone modifications to localize accessible chromatin regions that possibly influence the temporal gene transcription in the SCN. With the power of the definitive H3K27ac and H3K4me3 marks, we precisely located active enhancers and gene promoters in the SCN. Furthermore, comparative assessment of the histone modifications between the SCN and cortex helped us to identify tissue-specific active enhancers and promotors and answer the longstanding question about the exclusivity of the SCN. As a result, we clearly noted the abundance of tissue-specific H3K4me3-bound gene promoters (Supplemental Table S1) and H3K27ac-bound enhancers (Fig. 1D,E; Supplemental Table S2) around TSSs of the genes reported to be highly expressed in the central clock (Boguski and Jones 2004; Clark et al. 2013; Brown et al. 2017), such as the SCN-specific *Six6* gene (Supplemental Fig. S2C). This led us to produce the SCN regulatory map, highlighting the active enhancer sites that distinguishes SCN from the other brain region(s). These SCN-enriched active enhancer sites were found to be in close proximity of the genes associated with not just circadian entrainment but also calcium signaling and neuropeptidergic synaptic function (Fig. 1F), encompassing biological processes that support the daily timekeeping mechanism in the central clock. Consequently, the SCN-enriched enhancers were also seen adjacent to the genes defining SCN cell types such as neurons, astrocytes, and ependyma (Wen et al. 2020; Xu et al. 2021) and hold potential to further delimit the subtypes based on the regulatory elements.

Next, we wanted to explore if any of the identified SCN-enriched regulatory sites show changes in histone acetylation and/ trimethylation abundance with respect to time of the day. To this end, we examined the intensity of histone modifications in both the SCN and cortex at ZT3 (day) and ZT15 (night) and compared the resulting peak profiles. We clearly observed a significant proportion of varying H3K27ac peak abundance between day and night exclusively in the SCN (Supplemental Fig. S3B), with no difference in the cortex. This strongly suggests SCN-specific *cis*-elements that confer daily change in chromatin accessibility, potentially to control downstream rhythmic gene transcription. It clearly shows the importance of temporal control in genome-wide histone modifications within distinct brain regions in the context of daily timekeeping. The differential intensity noted in histone modification in the SCN was only observed for H3K27ac, and not for the H3K4me3 mark, which is profoundly enriched around the gene TSS. A plausible explanation for this phenomenon could be the occurrence of H3K4me3 marks around both poised and active gene TSSs (Beacon et al. 2021), whereas H3K27ac is reported to be present chiefly around active enhancers (Creyghton et al. 2010). It is tempting to speculate that histone trimethylation does not seem to be robustly changing and influencing temporal gene expression in response to environmental (such as light) cues in the SCN. Therefore, in our current study, H3K27ac was a better marker of dynamic changes, corroborating the notion that active enhancers are reliable determinants of spatiotemporally changing gene expression, which is a prerequisite for circadian timekeeping (Panigrahi and O'Malley 2021).

To explore further the occupancy of H3K27ac-defined active enhancers in the SCN, we assessed their abundance at varied 12-h separated time intervals (Supplemental Fig. S3C), and found robust 24-h oscillation (Fig. 4). Although this is consistent with the previously reported circadian modulation in histone modification levels observed in the peripheral clocks (Koike et al. 2012; Le Martelot et al. 2012), in our study we went a step further to assign transcripts that are under the control of these dynamic enhancers in the SCN. To address this, we executed bulk RNA-seq in parallel to histone ChIP sequencing at six distinct times of the day and noticed a modest correlation between fluctuating H3K27ac levels and neighboring rhythmic gene transcripts. On close inspection, the phase of peak H3K27ac abundance was often seen to precede the rising mRNA levels (Fig. 4F), consistent with the view that chromatin accessibility is offered by histone modification before achieving the required transcriptional burst (Karlić et al. 2010; Cheng and Gerstein 2012). This temporal relationship between the rhythmic H3K27ac and target gene expression levels varied on gene-by-gene basis, wherein in certain cases like *Nr4a3*, we noted H3K27ac peak ∼1–3 h preceding mRNA, whereas in *Phyhipl*, it was seen ∼4 h. Furthermore, with the help of the current transcriptional data set; we noticed that the peak of rhythmic gene expression, particularly for the core-clock genes, comes first in the SCN followed by its increase in other studied peripheral tissue(s) (Yamamoto et al. 2004; Abe et al. 2022). For example, *Per2* gene expression peaks around ZT8 (middle to late day) in the SCN, whereas it is reported to attain highest level around ZT14 (early night) in the liver. Hence, the systematic SCN transcriptomics conducted in the present study reconciled with the prior studies (Panda et al. 2002; Hogenesch et al. 2003) and offered an excellent opportunity to understand the relationship between changing chromatin state and gene expression.

Given the dynamic nature of histone acetylation in the SCN, we investigated the abundance of noncoding eRNAs that can regulate target gene expression, for instance, by mediating the formation of enhancer–promoter loops (Kim et al. 2015). These transcribing enhancers have also been recently discovered in multiple neuronal populations, as reported near the activity-induced *Fos* gene (Carullo et al. 2020), and found to be essential for induction of the target mRNA transcription. Thus, we capitalized on the exclusive bidirectional nature of eRNA transcription (Sartorelli and Lauberth 2020) and successfully identified a distinctive repertoire of actively transcribing eRNAs in the SCN. This was particularly challenging with regard to the size of the SCN and the limited amount of genomic material harvested from it. Nonetheless, with the high sequencing depth and biological replicates, we confidently showed the prevalence of bidirectionally transcribing noncoding eRNAs alongside H3K27ac in the SCN. A significant proportion of these noncoding eRNAs possessed robust daily oscillation in their expression levels, concomitant with the respective H3K27ac intensities, and appeared to control the rhythmic target mRNA expression in the SCN. The temporal relationship between the three endpoints, however, did not follow a defined trend and was noted to be locus specific (Supplemental Table S14). Moreover, at certain loci, we also observed rhythmic eRNAs arising from differential (not rhythmic) H3K27ac levels that point toward the possibility of histone acetylation being in charge of the chromatin accessibility (open or closed chromatin), whereas the corresponding eRNAs modulate the downstream target gene transcription. Nonetheless, our current finding is an extension to the formerly studied role of noncoding RNA (Mosig and Kojima 2022) such as miRNA (Xue and Zhang 2018; Zhou et al. 2021) in fine-tuning circadian clock by influencing the phase, amplitude, and period of the rhythm as noted across multiple organisms. Taken together, our pioneering efforts shed light on the prevalence of cycling *cis*-regulatory elements in the master pacemaker and clearly show its potential role in controlling gene expression machinery in the SCN.

This interwoven network of gene regulatory elements provides a hierarchical system of control over gene expression in the SCN. The dynamic change in histone acetylation followed by eRNA transcription lays the foundation for the coherent gene expression imperative for a daily timekeeping mechanism. Our present findings provide an excellent explanation how sequential layers of pretranscriptional regulation offer timely synchronization to the SCN transcriptional grid (Fig. 7). It also opens up the opportunity for further research on functional relationships between the molecular clock and epigenetic programming. Hence, it will be of interest to determine if genetic variation within any of these regulatory elements presents a threat to the well-orchestrated circadian timekeeping mechanism directed by the SCN. This could potentially constitute starting points to understand the association between aberrant gene-regulatory regions and the human disease pathologies, such as psychiatric and neurodegenerative disorders, resulting from circadian misalignment (Zhang et al. 2014; Logan and McClung 2019; Fagiani et al. 2022).

## Methods

### Mice

All animal studies were performed under the guidance issued by Medical Research Council in Responsibility in the Use of Animals for Medical Research (July 1993) and Home Office Project License 19/0004. WT C57BL/6J were maintained and provided in-house by MRC Harwell. Animals were group-housed (four to five mice per age) in individually ventilated cages under 12-h/12-h light–dark conditions with food and water available ad libitum.

### Experimental design

WT C57BL/6J mice (males, aged between 8 and 12 wk) were used for SCN tissue collection as described by Jagannath et al. (2013) at six distinct time points, starting from ZT3, at every 4 h, with lights on at 7 am (ZT0) and lights off at 7 pm (ZT12). We also collected cortical punches at ZT3 and ZT15 to compare and establish SCN-enriched chromatin modifications. An approximately 1-mm-thick mouse brain slice was sectioned between Bregma −0.1 and −1.0 mm using a brain matrix (Kent Scientific) and sterilized razor blades. The dissected mouse brain slice was placed on a cold block and promptly checked under a light microscope for the SCN (Bregma −0.3 and −0.8 mm, rostral to caudal) using cell-density contrast. Thereafter, the SCN (and cortex) was collected using a sample corer (1-mm internal diameter, Fine Science Tools) from the brain slice, flash-frozen on dry ice, and stored at −80°C. For histone ChIP, two separate biological replicates per time point per tissue type were collected, where each biological replicate comprised three to four individual SCN or cortical punches. For SCN-bulk RNA-seq, four biological replicates per time point were collected. Likewise, each biological replicate constituted three to four individual SCN samples.

### Histone ChIP and sequencing

ChIP was conducted with Diagenode ChIP-seq/ChIP-qPCR profiling service (Diagenode G02010000). Briefly, the chromatin was prepared using the true MicroChIP kit (Diagenode C01010130). Samples were fixed with 1% FA for 8 min. Chromatin was sheared using a Bioruptor Pico sonication device (Diagenode B01060001) combined with the Bioruptor water cooler for seven cycles, using 30-sec/30-sec on–off settings. Then, shearing was performed in 0.65-mL Bioruptor Pico microtubes (Diagenode C30010011). Twenty-five microliters of this chromatin was used to assess the size of the DNA fragments obtained by a high-sensitivity NGS fragment analysis kit (DNF-474) on a fragment analyzer (Advanced Analytical Technologies). ChIP was performed using a IP-Star compact automated system (Diagenode B03000002) following the protocol of the aforementioned kit. Five hundred nanograms of chromatin was immune-precipitated using 1 µg of each of the following Diagenode antibodies: H3K4me3 (C15410003; lot no. A1051D), H3K27ac (C15410196; lot no. A1723-0041D), and rabbit IgG (C15410206; lot no. RIG001). Chromatin corresponding to 1% was set apart as input. The DNA after reverse-cross-linking was quantified using a Qubit dsDNA HS assay kit (Thermo Fisher Scientific Q32854). Moreover, qPCR analysis was made to check ChIP efficiency using KAPA SYBR FAST (Sigma-Aldrich) on a LightCycler 96 system (Roche), and results were expressed as % recovery = 100 × 2^((Ct_input-6.64) − Ct_sample). Primers used were the following: the promoter of *Gapdh* (GAPDH-TSS) and myoglobin exon 2 (MBex2).

H3K4me3 and H3K27ac immune-precipitated genomic DNA along with their corresponding input samples was sent to Oxford Genomics Centre, University of Oxford, for library preparation using ChIP-seq protocol and paired-end sequencing on NovaSeq 6000 platform (Illumina).

### Histone ChIP-seq mapping and peak calling

Raw sequence data in the form of a pair of fq.gz were processed using tools on the Galaxy EU server (Afgan et al. 2016; https://usegalaxy.eu/) using the ChIP-seq pipeline. Paired-end FASTQ files were quality assessed by removing low-quality bases (Phred < 20) and trimmed using FastQC (https://www.bioinformatics.babraham.ac.uk/projects/fastqc/) and Trimmomatic v0.36 (http://www.usadellab.org/cms/index.php?page=trimmomatic), respectively. FASTQ files containing trimmed sequences were then aligned to the mm10 genome assembly to generate binary alignment map (BAM) files with Bowtie 2 v2.3.4.1 (Langmead et al. 2009). Aligned files were filtered for minimum mapping quality (MAPQ) > 20 by SAMtools v1.8 (https://samtools.github.io/hts-specs/) (Li et al. 2009) and used for peak calling by MACS2 v2.1.1.20160309 (Feng et al. 2012) with the options ‐‐qvalue 0.05 ‐‐gsize mm:1.87e9 ‐‐format BAMPE. The plotPCA function of deepTools2 suite (Ramírez et al. 2016) was used with the default (top 1000 most variable rows) settings to conduct an initial quality analysis through PCA on the enrichment for H3K4me3 and H3K27ac. A matrix file containing genomic regions split into 10,000 bin size as rows and sample BAM files (read coverage) per column was generated by multiBamSummary and used as an input file to compute PCA. Finally, peaks for each brain region and histone modification (H3K4me3, H3K27ac) were analyzed for differential binding by a Bioconductor package Diffbind v2.10.0 (http://bioconductor.org/packages/release/bioc/vignettes/DiffBind/inst/doc/DiffBind.pdf).

### Bulk RNA-sequencing

Bulk RNA-sequencing (RNA-seq) was performed at Oxford Genomics Centre, University of Oxford. RNA was extracted, DNase-treated, and purified (RNeasy, Qiagen) for four biological replicates per time point. Five hundred nanograms to 1 µg of total RNA underwent quality control (NanoDrop), and libraries were prepared for directional ribodepleted RNA-seq using NEBNext reagents. RNA-seq libraries underwent sequencing (150-bp paired-end directional reads: approximately 50 million reads/sample) on a NovaSeq 6000 (Illumina) platform.

### Bulk RNA-seq data analysis

Paired-end FASTQ files were quality assessed (Phred < 20 removed) with FastQC (https://www.bioinformatics.babraham.ac.uk/projects/fastqc/), and Illumina adapters were trimmed with Trim Galore! v0.4.3 (https://www.bioinformatics.babraham.ac.uk/projects/trim_galore/). Then the reads were aligned to the mm10 genome assembly using STAR v2.7.8a (Dobin et al. 2013) with the MAPQ value for unique mappers set to 60. BAM files were used to generate read counts per gene by featureCounts (Liao et al. 2014) via SAMtools v1.11. Finally, the limma-voom method (Liu et al. 2015) from the Bioconductor package-limma v3.48.0 (http://bioconductor.org/packages/release/bioc/html/limma.html) was adopted to quantify differential gene expression, and normalized logarithmic CPM values were generated for downstream analysis.

### eRNA identification

Enhancer identification was performed by investigating H3K27ac sites as described above. MACS2-defined and Diffbind-identified differential H3K27ac peaks coupled with total RNA-seq BAM files were used to predict bidirectional eRNA transcription using Seqmonk software (https://www.bioinformatics.babraham.ac.uk/projects/seqmonk/) as previously reported (Carullo et al. 2020). Briefly, for eRNA identification, we focused on the intergenic H3K27ac enrichment sites as intragenic sites posed a risk of alternate promoters and other regulatory elements plus nascent gene transcription. Any differential intergenic H3K27ac site that showed bidirectional transcription was further assessed for eRNA–target gene pairs. These were identified by mapping gene promoters within 2 Mb upstream of or downstream from the center of the peak. CPKM values for each identified eRNA and associated gene were correlated using Pearson's correlation in R (R Core Team 2022). eRNA–gene pairs with correlations > 0.5 were considered as high-confidence pairs.

### HOMER motif analysis

Differential region– or time point–specific genomic positions were compiled into BED files. The findMotifsGenome.pl function within the HOMER v4.11 (Heinz et al. 2010) package was used to identify enriched motifs and their corresponding TFs with options size 1000 –len 8,10,12 –mask –preparse –dumpfasta. For interested region-specific motifs such as SCN, genomic BED files from the cortical region were used as the background and vice versa.

### Gene annotation

The gene list derived from Bioconductor-based ChIPseeker v1.28.3 (Yu et al. 2015) was fed into the Database for Annotation, Visualization, and Integrated Discovery (DAVID) tool (Dennis et al. 2003). The functional annotation chart based on KEGG pathway and Gene Ontology (GO::BP) was plotted with the help of the ggplot 2 package in R v4.0.5 (https://ggplot2.tidyverse.org/).

### Analysis of oscillating H3K27ac signals, genes and eRNAs

Logarithmic CPM values across all time points for H3K27ac occupancy, gene, and eRNA feature were analyzed for significant circadian oscillations using JTK_CYCLE (Wu et al. 2016) and ECHO (De Los Santos et al. 2020). For H3K27ac oscillation, significantly differential H3K27ac sites between any two antiphasics (12 h apart) were compiled using BEDTools v2.30.0 (Quinlan and Hall 2010). Normalized CPM values from compiled 1021 intervals were used for 24-h rhythmicity assessment.

### RNA extraction and RT-qPCR

Total RNA was extracted from the SCN tissue as described above at three distinct time points; ZT3, ZT15, and ZT23. For each time point, RNA was extracted from two to four biological replicates, where each biological replicate comprises three to four individual SCN punches. One hundred nanograms RNA per sample replicate was reverse-transcribed (iScript cDNA synthesis kit, Bio-Rad; 5 min at 25°C, 20 min at 46°C, 1 min at 95°C, hold at 4°C). The synthesized cDNA was used for RT_qPCR using an Applied Biosystems 7500 real-time PCR instrument (SsoAdvanced universal SYBR Green supermix, Bio-Rad; 1 μL cDNA in 20 μL, 30 sec at 95°C, 40× 15 sec at 95°C, and 60 sec at 60°C, followed by melt curve analysis 65°C–95°C in 0.5°C increments at 5 sec per step) in triplicates for the genes of interest (*Vipr1*, eRNA; Chr 9: 123,334,375–123,335,717). 7500 Applied Biosystems Software v2.3 (https://www.thermofisher.com/uk/en/home/technical-resources/software-downloads/applied-biosystems- 7500-real-time-pcr-system.html) was used to obtain the relative quantification values and examine melt curves. All RT-qPCR data were normalized to the *Rpl32* reference control and analyzed using the standard curve method (Larionov et al. 2005) The primers used for *Vipr1* (TCAACAACGGGGAGACAGAC [forward] and GGCCATGACGCAATACTGGA [reverse]), eRNA (TGGTTAAGAGCGCCTACAGC [forward] and GCTGTCTTCAGACACTCCAGA [reverse]), and *Rpl32* (AGGCACCAGTCAGACCGATA [forward] and TGTTGGGCATCAGGATCTGG [reverse]) were designed and screened for target specificity with the National Center for Biotechnology Information's (NCBI) Basic Local Alignment Search Tool (BLAST) (https://blast.ncbi.nlm.nih.gov/). The primer sets were validated before use (requirements: no predicted off-targets, primer efficiency 85,120%, no significant signal in nontemplate control, single peak in melt curve, and single band at the predicted size when separated via agarose gel electrophoresis).

### Statistical analysis

Gene expression differences from qPCR were plotted and analyzed with GraphPad Prism v9.5.1 for Windows, GraphPad Software (www.graphpad.com). The Pearson's correlation between the target mRNA and eRNA across the tested time points was set at alpha = 0.05.

## Data access

All raw and processed sequencing data generated in this study have been submitted to the NCBI Gene Expression Omnibus (GEO; https://www.ncbi.nlm.nih.gov/geo/) under accession number GSE217943.

## Supplementary Material

Supplemental Material
