# Supplementary material for: Dynamic modulation of genomic enhancer elements in the suprachiasmatic nucleus, the site of the mammalian circadian clock
Source: Genome Res. 2023 May;33(5):673–88. doi: 10.1101/gr.277581.122 (PMC10317116; doi:10.1101/gr.277581.122)
Supplement: Supplemental Material [file supp_33_5_673__DC1.html]

Dynamic modulation of genomic enhancer elements in the suprachiasmatic nucleus, the site of the mammalian circadian clock — Supplemental Material 

# Dynamic modulation of genomic enhancer elements in the suprachiasmatic nucleus, the site of the mammalian circadian clock

## Supplemental Material

- Supplemental\_Fig\_S1.pdf
- Supplemental\_Fig\_S2.pdf
- Supplemental\_Fig\_S3.pdf
- Supplemental\_Fig\_S4.pdf
- Supplemental\_Fig\_S5.pdf
- Supplemental\_Fig\_S6.pdf
- Supplemental\_Fig\_S7.pdf
- Supplemental\_Table\_S1.xlsx
- Supplemental\_Table\_S2.xlsx
- Supplemental\_Table\_S3.xlsx
- Supplemental\_Table\_S4.xlsx
- Supplemental\_Table\_S5.xlsx
- Supplemental\_Table\_S6.xlsx
- Supplemental\_Table\_S7.xlsx
- Supplemental\_Table\_S8.xlsx
- Supplemental\_Table\_S9.xlsx
- Supplemental\_Table\_S10.xlsx
- Supplemental\_Table\_S11.xlsx
- Supplemental\_Table\_S12.xlsx
- Supplemental\_Table\_S13.xlsx
- Supplemental\_Table\_S14.docx
