# Supplementary material for: Dynamic modulation of genomic enhancer elements in the suprachiasmatic nucleus, the site of the mammalian circadian clock
Source: Genome Res. 2023 May;33(5):673–88. doi: 10.1101/gr.277581.122 (PMC10317116; doi:10.1101/gr.277581.122)
Supplement: Supplemental Material [file supp_gr.277581.122_Supplemental_Fig_S1.pdf]

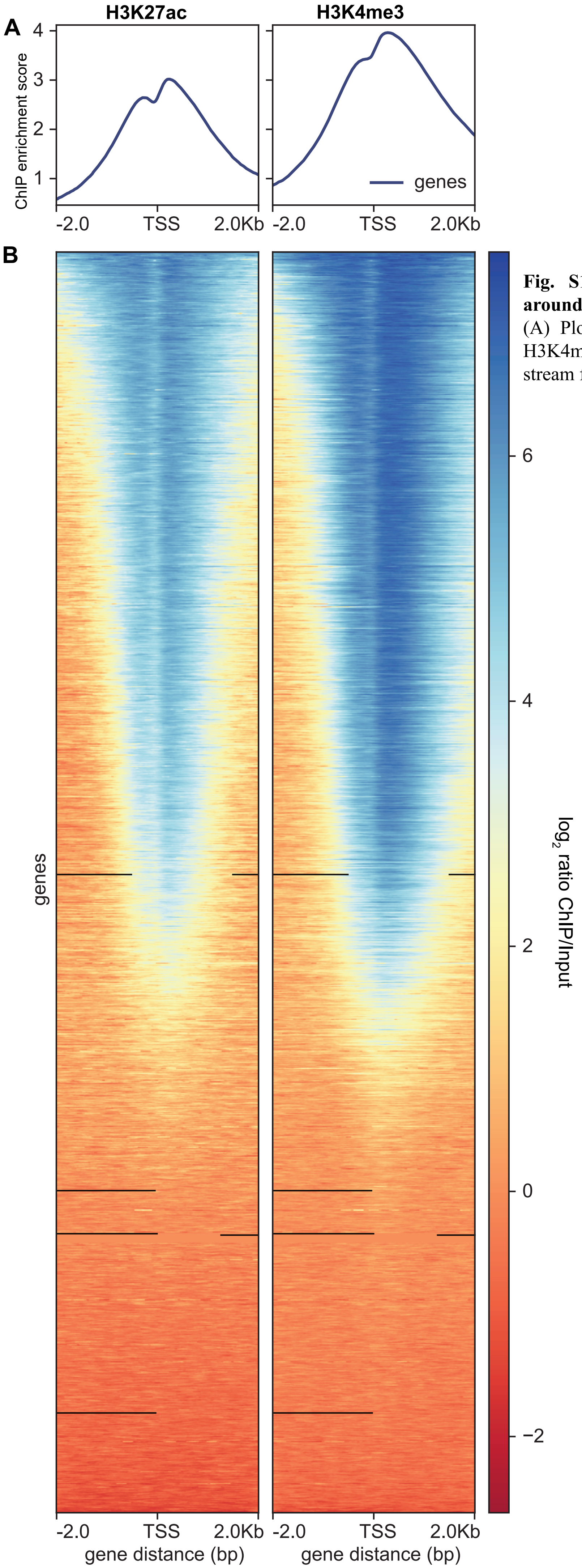

Fig. S1:Distribution of H3K4me3 and H3K27ac sites around TSS.
(A) Plot profile and (B) Heatmap showing distribution of H3K4me3 and H3K27ac sites up to 2 kb upstream and downstream from transcription start site (TSS).
