# Supplementary material for: Dynamic modulation of genomic enhancer elements in the suprachiasmatic nucleus, the site of the mammalian circadian clock
Source: Genome Res. 2023 May;33(5):673–88. doi: 10.1101/gr.277581.122 (PMC10317116; doi:10.1101/gr.277581.122)
Supplement: Supplemental Material [file supp_gr.277581.122_Supplemental_Fig_S2.pdf]

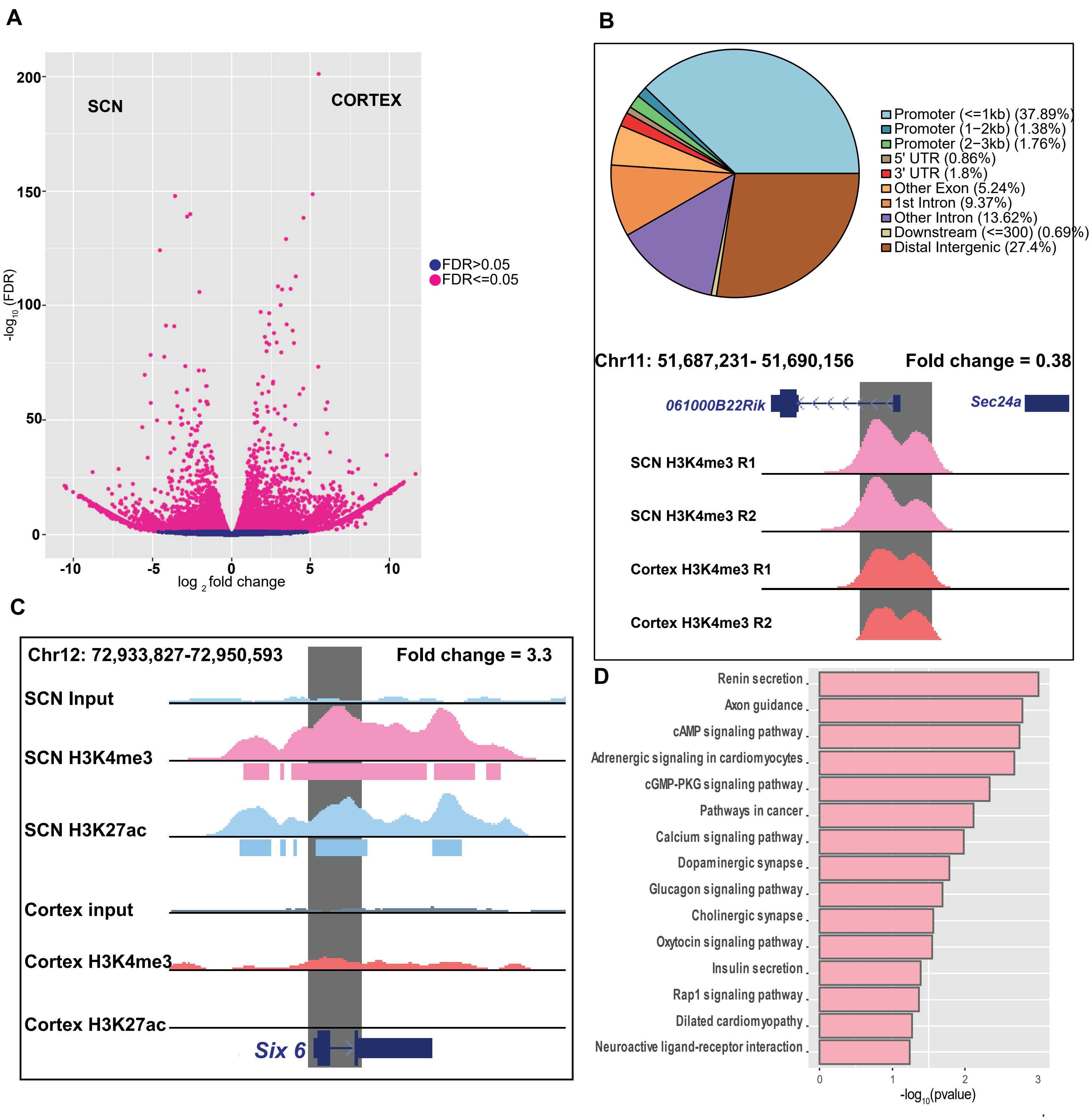

Fig. S2. Differential H3K4me3 sites between SCN and Cortex. (A)Volcano plot showing fold change and false discovery rate (FDR) for differential H3K4me3 sites between SCN and cortex as computed by Diffbind. (B) Top:Genomic feature distribution of differential H3K4me3 sites between SCN and cortex (n = 10577), Below: An example of UCSC Genome Browser tracks showing differential H3K4me3 occupancy (normalized ChIP-seq coverage) between SCN and Cortex at promoter.(C) UCSC Genome Browser tracks showing H3K4me3 and H3K27ac normalized ChIP-seq coverage and peaks around *Six6* locus (shaded grey), demonstrating H3K4me3 enrichment in SCN (pink vs red genome coverage). (D) Functional annotation of nearest neighbouring gene (TSS) to SCN enriched H3K4me3 sites (fold change > 5) using KEGG pathway (DAVID)
