# Supplementary material for: Dynamic modulation of genomic enhancer elements in the suprachiasmatic nucleus, the site of the mammalian circadian clock
Source: Genome Res. 2023 May;33(5):673–88. doi: 10.1101/gr.277581.122 (PMC10317116; doi:10.1101/gr.277581.122)
Supplement: Supplemental Material [file supp_gr.277581.122_Supplemental_Fig_S3.pdf]

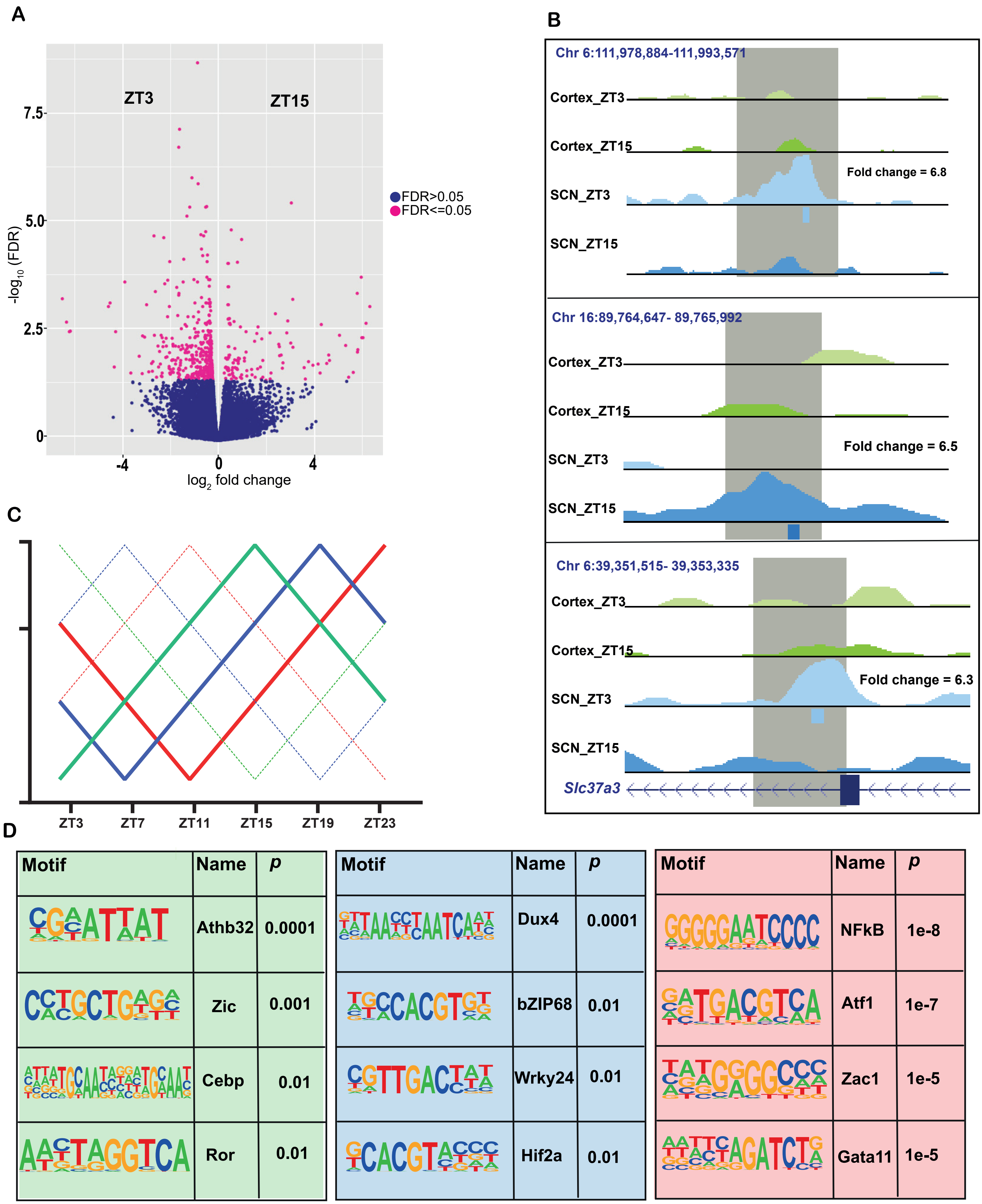

Fig. S3 Differing H3K27ac occupancy at anti-phasic timepoints. (A)Volcano plot showing fold change and false discovery rate (FDR) for differential H3K4me3 sites between ZT3 and ZT15 (n = 551) in cortex. (B) UCSC Genome Browser images of three separate genomic regions showing differential ZT3 Vs ZT15 H3K27ac abundance specifically in SCN , fold change and chromosome positions indicated per region. (C) Representative model illustrating anti-phasic timepoint comparisons with bold and dashed lines for ZT3 Vs ZT15 (green), ZT7 Vs ZT19 (blue) and ZT11 Vs ZT23 (red). (D) Enriched motif at differential H3K27ac sites observed between ZT3 Vs ZT15 (green shaded), ZT7 Vs ZT19 (blue shaded) and ZT11 Vs ZT23 (red shaded).
