# Supplementary material for: Dynamic modulation of genomic enhancer elements in the suprachiasmatic nucleus, the site of the mammalian circadian clock
Source: Genome Res. 2023 May;33(5):673–88. doi: 10.1101/gr.277581.122 (PMC10317116; doi:10.1101/gr.277581.122)
Supplement: Supplemental Material [file supp_gr.277581.122_Supplemental_Fig_S4.pdf]

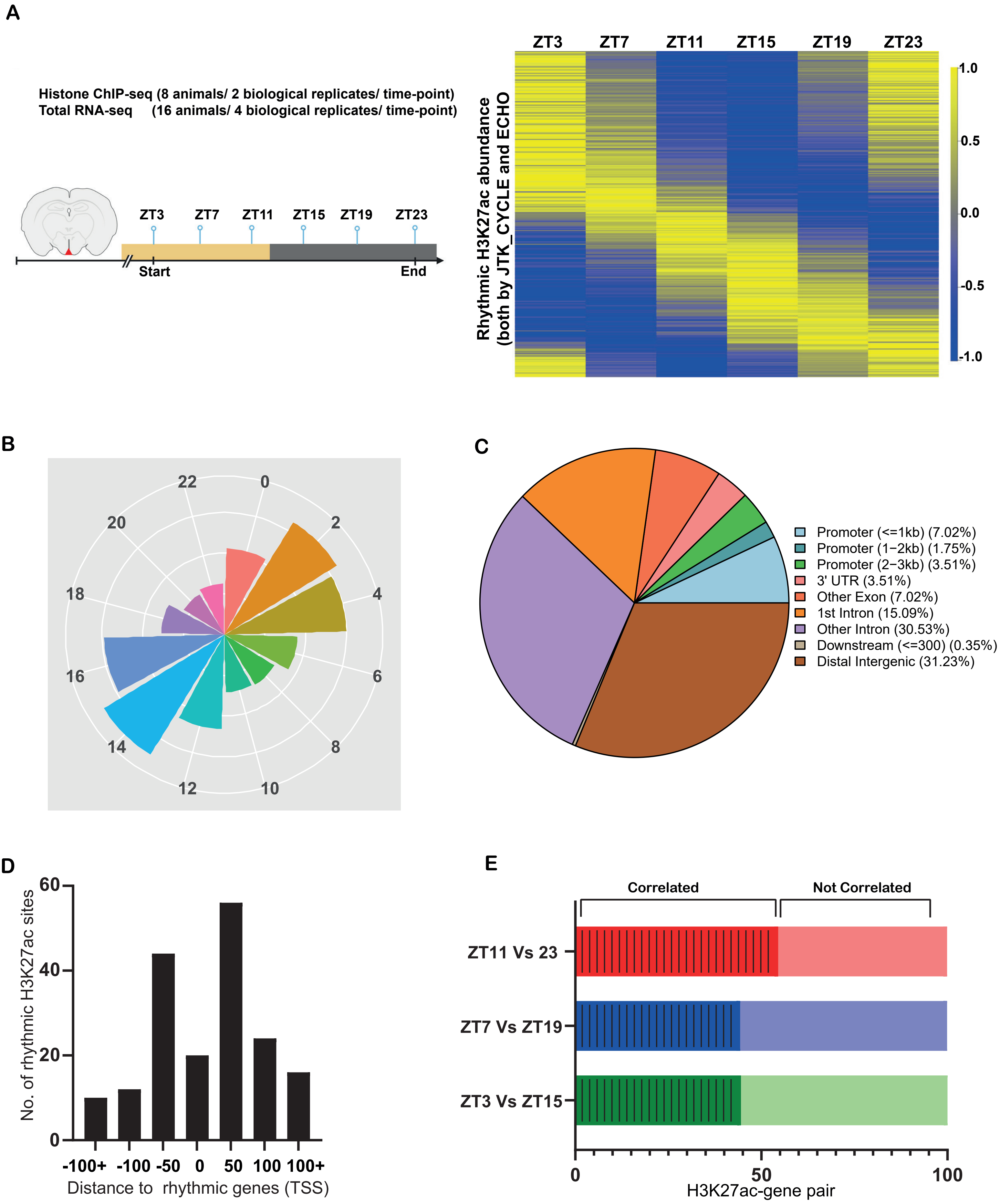

Fig. S4 **Diurnal variation in H3K27ac occupancy.** (A) Illustration of SCN tissue collection for histone ChIP-seq and RNA-seq at six distinct time-points and heatmap showing rhythmic (both ECHO and JTK_CYCLE) abundance of H3K27ac across the day. (B) Rose plot showing phase (peak expression) distribution (12 groups) of rhythmic genes in SCN. The area of each wedge is proportional to number of rhythmic genes in that group. (C) Genomic feature distribution of rhythmic H3K27ac intervals as analysed by ChIPseeker. (D) Bar plot indicating the number of rhythmic H3K27ac sites and their distance from closest rhythmic gene/TSS (kb). (E) Proportion of correlated (Pearson's correlation = 1) and not correlated (Pearson's correlation = -1) H3K27ac- gene levels for each compared antiphasic time-points.
