# Supplementary material for: Dynamic modulation of genomic enhancer elements in the suprachiasmatic nucleus, the site of the mammalian circadian clock
Source: Genome Res. 2023 May;33(5):673–88. doi: 10.1101/gr.277581.122 (PMC10317116; doi:10.1101/gr.277581.122)
Supplement: Supplemental Material [file supp_gr.277581.122_Supplemental_Fig_S5.pdf]

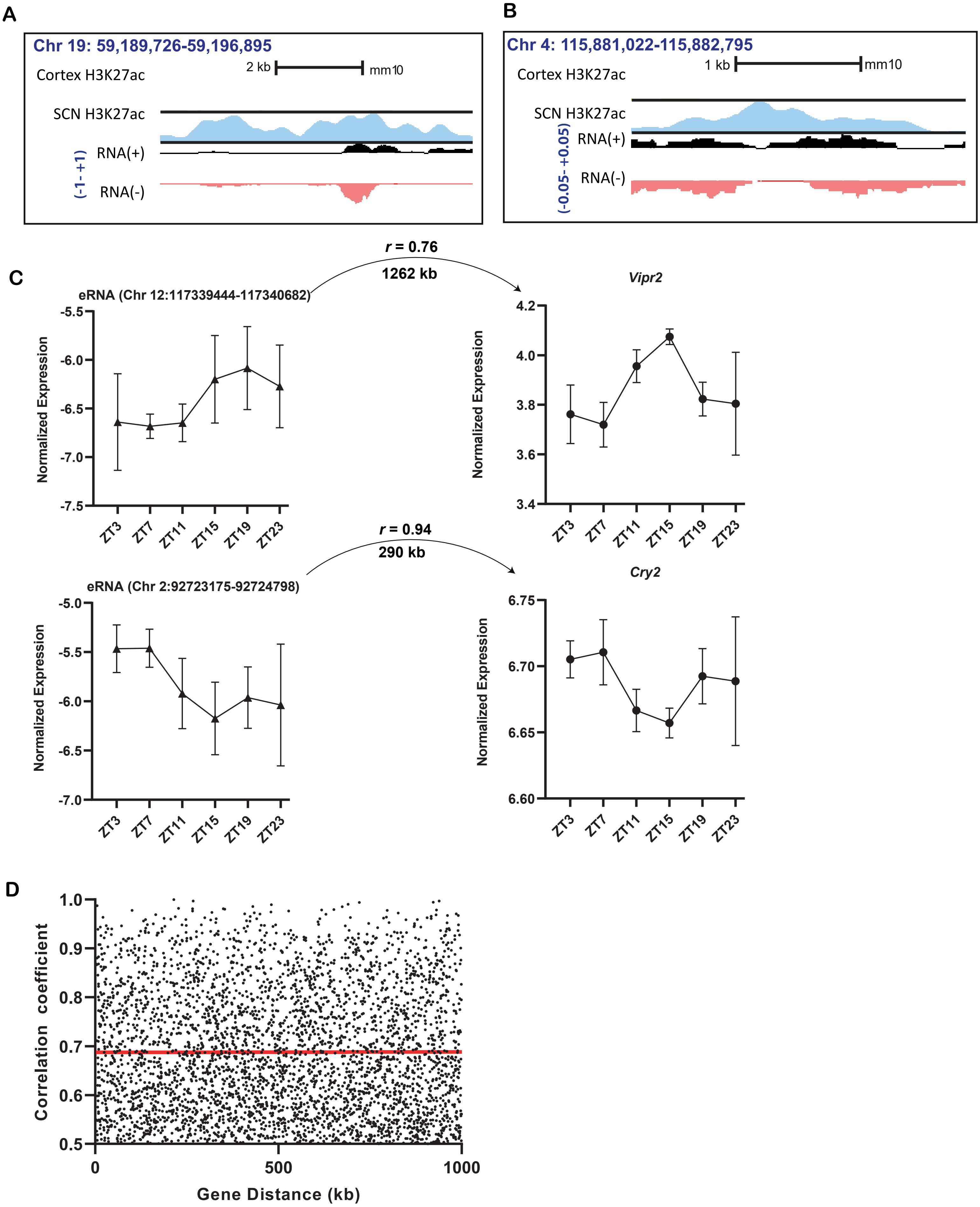

Fig. S5. Enhancer RNA identification in SCN. (A and B) Genome Browser images showing bidirectional transcription (signal as normalized CPM value from + (black) and - (red) RNA strands) arising from H3K27ac marked SCN enhancer peak (blue), at marked mm10 chromosome coordinates. (C) Normalized expression levels of eRNA and target mRNA for two representative examples i.e. *Vipr2, Cry2.* Distance between eRNA and predicted target gene and Pearson's correlation is indicated above each representated eRNA-gene pair. (D) Scatter plot showing no relation between gene distance and strength of correlation ($r > 0.5$) as indicated by red line.
