# Supplementary material for: Dynamic modulation of genomic enhancer elements in the suprachiasmatic nucleus, the site of the mammalian circadian clock
Source: Genome Res. 2023 May;33(5):673–88. doi: 10.1101/gr.277581.122 (PMC10317116; doi:10.1101/gr.277581.122)
Supplement: Supplemental Material [file supp_gr.277581.122_Supplemental_Fig_S6.pdf]

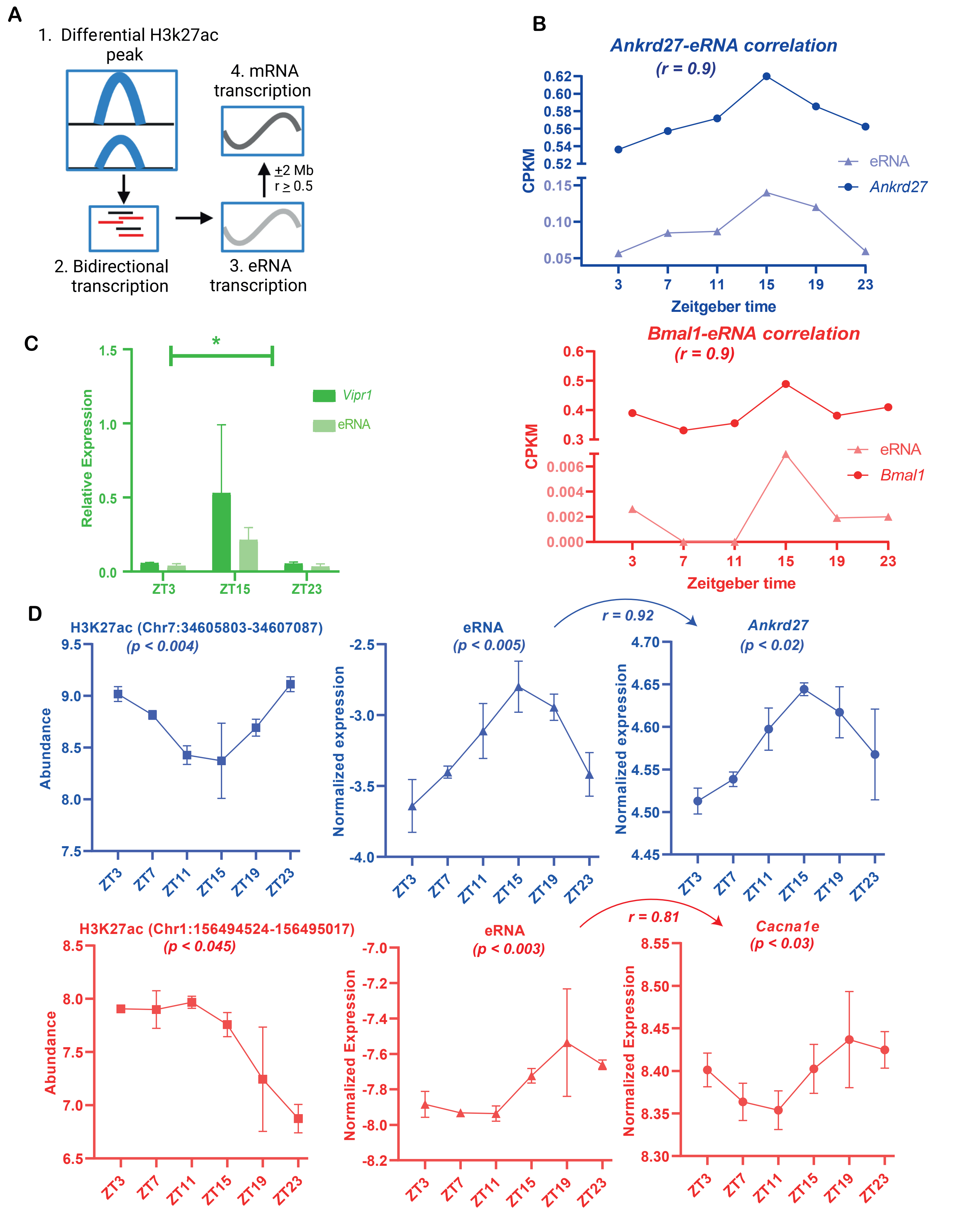

Fig. S6 Differential H3K27ac abundance linked with eRNA and target mRNA transcription. (A)Schematic of eRNA identification from differential H3K27ac peaks (B) eRNA and positively correlated ($r > 0.5$) target mRNA expression (CPKM) during the day. (C) Relative expression of eRNA (Chr9: 123334375-123335717) and *Vipr1* mRNA analyzed by qPCR at ZT3, 15 and 23, showed perfect correlation in peak and trough expression levels (Pearson's correlation (r) = 0.99, $p < 0.01$ as denoted by *). (D) Representative examples of differential H3K27ac sites (chromosome coordinates indicated at top) observed across ZT7 Vs ZT19 (blue) and ZT11 Vs ZT23 (red), showing rhythmic H3K27ac abundance along with cyclic eRNA and target gene expression (log CPM) as analysed by ECHO ($p<0.05$) with indicated Pearson's correlation coeeficient (r).
