# Supplementary material for: Dynamic modulation of genomic enhancer elements in the suprachiasmatic nucleus, the site of the mammalian circadian clock
Source: Genome Res. 2023 May;33(5):673–88. doi: 10.1101/gr.277581.122 (PMC10317116; doi:10.1101/gr.277581.122)
Supplement: Supplemental Material [file supp_gr.277581.122_Supplemental_Fig_S7.pdf]

A

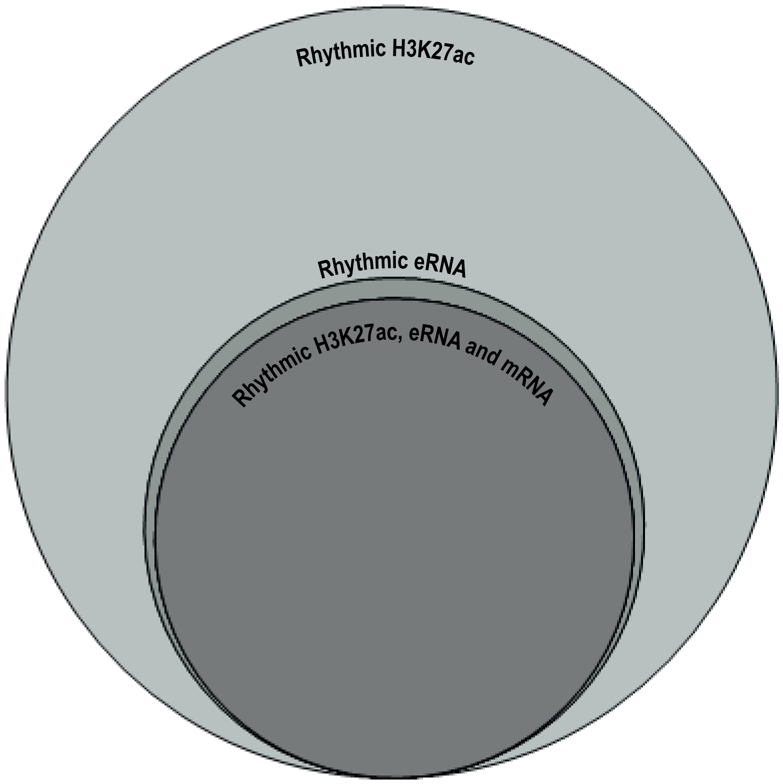

B

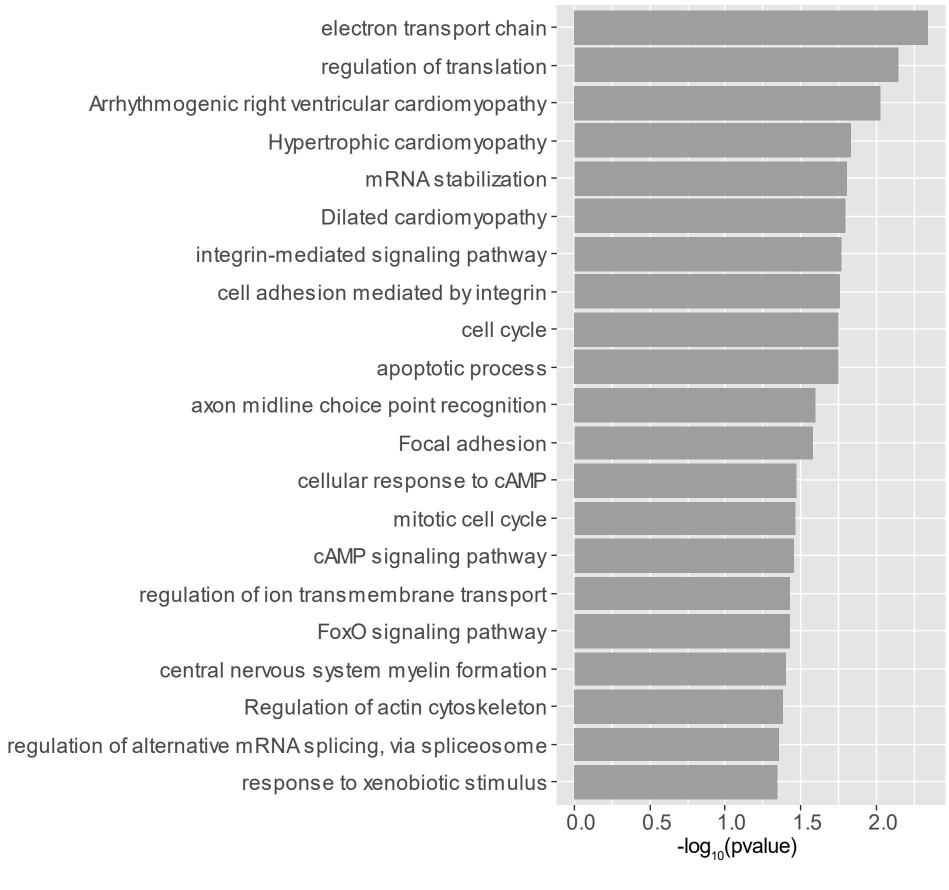

Fig. S7. **Rhythmic H3K27ac linked with cyclic eRNA and target mRNA transcription.** (A) Stacked venn diagram showing proportion of rhythmic eRNA (42%) and cyclic mRNA (39%) overlapping with rhythmic H3K27ac sites present at intergenic loci.(B) Functional annotation of cyclic genes linked to rhythmic eRNA and H3K27ac using gene ontology by BP (biological process) and KEGG pathway (DAVID).
