# Supplementary material for: Dynamic modulation of genomic enhancer elements in the suprachiasmatic nucleus, the site of the mammalian circadian clock
Source: Genome Res. 2023 May;33(5):673–88. doi: 10.1101/gr.277581.122 (PMC10317116; doi:10.1101/gr.277581.122)
Supplement: Supplemental Material [file supp_gr.277581.122_Supplemental_Table_S14.docx]

| **Chr** | **Start** | **End** | **H3K27ac_phase** | **eRNA_phase** | **mRNA_phase** | **Gene** |
| --- | --- | --- | --- | --- | --- | --- |
| 9 | 48618390 | 48619728 | 0 | 2 | 2 | *Gm5617* |
| 7 | 34605803 | 34607087 | 0 | 14 | 14 | *Ankrd27* |
| 11 | 1.06E+08 | 1.06E+08 | 0 | 18 | 20 | *C130046K22Rik* |
| X | 11435051 | 11435879 | 2 | 6 | 6 | *1810030O07Rik* |
| 12 | 1.05E+08 | 1.05E+08 | 2 | 10 | 12 | *Papolb* |
| 2 | 1.74E+08 | 1.74E+08 | 2 | 12 | 12 | *Aurka* |
| 1 | 1.35E+08 | 1.35E+08 | 2 | 20 | 22 | *Epyc* |
| 5 | 1.21E+08 | 1.21E+08 | 4 | 6 | 4 | *Ddx54* |
| 5 | 65908754 | 65910741 | 4 | 10 | 4 | *Klb* |
| 8 | 10811516 | 10813188 | 6 | 6 | 6 | *Irs2* |
| 3 | 1.4E+08 | 1.4E+08 | 6 | 14 | 10 | *Unc5c* |
| 3 | 1.08E+08 | 1.08E+08 | 6 | 14 | 16 | *Cyb561d1* |
| 1 | 1.56E+08 | 1.56E+08 | 6 | 18 | 18 | *Cacna1e* |
| 6 | 89268755 | 89269315 | 8 | 0 | 0 | *H1f10* |
| 9 | 50118325 | 50119585 | 8 | 6 | 6 | *Rbm7* |
| 11 | 1.17E+08 | 1.17E+08 | 8 | 12 | 14 | *Afmid* |
| 11 | 88821136 | 88821452 | 8 | 16 | 16 | *2210416O15Rik* |
| 11 | 1.14E+08 | 1.14E+08 | 8 | 20 | 20 | *Cog1* |
| 16 | 85199602 | 85201391 | 10 | 0 | 0 | *Adamts1* |
| 10 | 21635645 | 21637787 | 10 | 10 | 10 | *Sgk1* |
| 12 | 51118348 | 51119376 | 12 | 6 | 6 | *Foxg1* |
| 1 | 1.8E+08 | 1.8E+08 | 12 | 12 | 12 | *Ahctf1* |
| 10 | 21650407 | 21655338 | 12 | 12 | 12 | *Hbs1l* |
| 7 | 74213056 | 74213740 | 12 | 18 | 20 | *Klhl25* |
| 12 | 12827247 | 12828811 | 14 | 10 | 14 | *Kcns3* |
| 2 | 25476515 | 25480954 | 14 | 12 | 12 | *Vav2* |
| 15 | 81834690 | 81840634 | 16 | 10 | 10 | *Mkl1* |
| 9 | 1.2E+08 | 1.2E+08 | 16 | 12 | 14 | *Trak1* |
| 9 | 1.23E+08 | 1.23E+08 | 16 | 14 | 14 | *Vipr1* |
| 3 | 98266269 | 98268526 | 16 | 16 | 14 | *Itga10* |
| 14 | 49127089 | 49127650 | 16 | 16 | 18 | *Peli2* |
| 17 | 53542641 | 53545178 | 16 | 16 | 22 | *Satb1* |
| 15 | 58705903 | 58707379 | 18 | 12 | 10 | *Trib1* |
| 18 | 60901342 | 60902420 | 22 | 12 | 12 | *Tcof1* |

**Supplemental Table S14**: Temporal relationship between rhythmic H3K27ac, eRNA and mRNA.
